# Antenna photosystem I remodeling in the iron stress–induced complex from *Anabaena* sp. PCC 7120

**DOI:** 10.1126/sciadv.aeg3567

**Published:** 2026-08-12

**Authors:** Ranel Maqdisi, Dariusz M. Niedzwiedzki, Christopher Gorski, Sandeep Biswas, Himadri B. Pakrasi, Yuval Mazor

**Affiliations:** ^1^ School of Molecular Sciences, Arizona State University, Tempe, AZ 85287-1004, USA.; ^2^ Department of Biology, Washington University, St. Louis, MO 63130, USA.

## Abstract

The structure and oligomeric states of the photosystem I–iron stress–induced protein A (PSI-IsiA) supercomplex from several species have been investigated using cryo–electron microscopy (cryo-EM) and atomic force microscopy. The cryo-EM structure of the PSI-IsiA complex from cyanobacterium *Anabaena sp. PCC 7120* (7120) has been published previously. However, not all IsiA and PSI subunits were identified. In this work, we have structurally and spectroscopically characterized the PSI-IsiA supercomplex from 7120 using single-particle cryo-EM at a resolution of 2.2 angstroms. The data resolved all individual IsiA and PSI subunits, highlighting features that distinguish the 7120 PSI-IsiA from other complexes, including additional IsiA and a unique PsaK subunit specific for PSI-IsiA. Time-resolved fluorescence spectroscopy showed tight energetic coupling of IsiA to PSI, which may play a role in lowering antenna fluorescence yield and increasing the efficiency of energy transfer. This work extends the scope of PSI cellular remodeling in response to different stress conditions.

## INTRODUCTION

Oxygenic photosynthesis is one of the most crucial biochemical processes on Earth. Not only is it considered the primary energy input into the global food chain, but it also largely determines the composition of Earth’s atmosphere (*1*). Phytoplankton such as cyanobacteria are responsible for producing most of the atmospheric oxygen content on Earth and a large proportion of the net primary production of organic matter (*2*). Alongside plants and algae, these organisms have the required machinery for performing photosynthesis, which is a composite of light reactions that convert sunlight into chemical energy and light-independent reactions that fix carbon dioxide into complex organic compounds.

Embedded in thylakoid membranes are several large protein complexes that make up the oxygenic photosynthetic electron transport chain including photosystem I (PSI), photosystem II (PSII), cytochrome b_6_f, and ATP synthase (*3*). It is estimated that the photosynthetic apparatus of oxygenic organisms is particularly rich in iron with an estimated total of 22 to 23 iron atoms per chain, half of which are allocated to iron-sulfur clusters of PSI (*4*, *5*). The iron quota of phototrophic and oxygenic cyanobacterium *Synechocystis* sp. *PCC 6803* (6803) is one order of magnitude higher than that of *Escherichia coli* (*6*). Moreover, the intracellular iron demand is usually much higher than its availability in the immediate environment (*7*). The oxidized ferric form of iron tends to form insoluble hydroxides in oxygenic and neutral pH aqueous habitats and is often bound by organic ligands, which reduces its bioavailability (*8*). Therefore, despite iron being the fourth most abundant element in Earth’s crust, deprivation of this nutrient has been observed to impose great limitations on biomass production of phytoplankton not only in oceans but also in coastal regions and lakes (*9*–*11*).

Cyanobacteria exhibit great diversity stemming from billions of years of adaptation to nearly every possible environment on Earth (*12*, *13*). Environmental stress can stem from light intensity, temperature fluctuations, and nutrient imbalances that enhance production of reactive oxygen species contributing to oxidative stress that damages biomolecules. At the level of protein structure and sequence, antenna systems exhibit greater diversity and versatility compared to the conserved reaction center core (*14*). Antenna proteins are densely packed with pigments and are optimized to harvest and transfer excitation energy into reaction centers (*15*). The iron stress–induced protein A (IsiA) is one of the few antenna systems known to exist in cyanobacteria alongside prochlorophyte chlorophyll a/b binding proteins (Pcbs) and phycobilisomes (*16*). Prolonged iron deprivation has been the major factor associated with IsiA expression, but up-regulation of the IsiA gene has been observed under heat, high salt, oxidative chemicals, and high light exposure (*17*–*20*). IsiA was initially identified in *Anacystis nidulans R2* (now known as *Synechococcus elongatus PCC 7942*) as a chlorophyll a (ChlA) binding protein homologous to CP43 (*21*, *22*). Later studies showed it to efficiently couple with PSI reaction center in both 6803 and *Synechococcus* sp. *PCC 7942*, forming supermolecular complexes of 18 subunit rings around a trimeric core (PSI_3_-IsiA_18_) (*23*–*28*). The IsiA/Pcb chlorophyll a/b binding protein family has a conserved core of six transmembrane helices (TMHs) (*29*). Moreover, these proteins have been implicated in serving a role in both light harvesting as antenna to PSI and photoprotection through excitation energy quenching (*29*–*31*).

PSI in cyanobacteria assumes different oligomeric forms, most commonly trimeric, the relevance of which is not fully understood but may serve as an adaptation to different light conditions (*32*, *33*). PSI is considered to be the most efficient light energy converter in nature with a quantum yield near unity (*33*). Among the various strains of cyanobacteria, the nitrogen-fixing *Anabaena* sp. *PCC 7120* (hereafter 7120) has been shown to have tetrameric, dimeric, and monomeric PSI forms (*34*, *35*). Similar to many cyanobacteria, the 7120 genome contains multiple IsiA genes (four genes) that have been shown to be transcribed in iron-limited growth conditions (*36*). In addition to the variability in the number of IsiA genes in cyanobacterial species, the protein shows intrinsic oligomeric versatility forming single, double, and multiple rings both with and without a PSI core (mainly shown in PSI_3_-IsiA_18_ species) (*37*, *38*). The formation of IsiA-only aggregates, a phenomenon that was thought to be a by-product of chlorosis (prolonged iron stress), has been associated with an early-onset photo-protective role during iron stress exhibiting unprecedented, shortened ChlA fluorescence lifetimes (*38*, *39*). Recently, cryo–electron microscopy (cryo-EM) structures of double-layered PSI-IsiA supercomplexes in thylakoid membranes were determined, giving further insight into the variability that exists in these systems and its role in adaptation to iron stress (*40*). It is evident that much remains unknown about the diversity of structure and function in this protein, with respect to its binding dynamics, mechanisms of oligomerization, and interactions with the photosynthetic machinery in thylakoid membranes.

The structure and oligomeric states of the membrane-embedded PSI-IsiA supercomplex have been a major hotspot for studies using cryo-EM and atomic force microscopy (*36*, *40*–*44*). The single-particle cryo-EM structure of the PSI-IsiA complex from 7120 has been determined at a resolution of 2.62 Å (*36*). The structure revealed a complex of six IsiA antenna around a monomeric PSI core including an IsiA-PsaL fusion (IsiA2) protein and other photosynthetic cofactors. Nevertheless, the resolution of the cryo-EM map prevented detailed modeling of some IsiAs as well as their interactions with PSI. In the current work, we have determined and spectroscopically characterized the structure of the PSI-IsiA supercomplex from 7120 using single-particle cryo-EM. The resulting 2.2-Å map revealed specific binding sites of all IsiA proteins in 7120, an additional IsiA subunit in this complex, in addition to a PSI-IsiA_7_ specific PsaK subunit and showed that individual IsiAs bind to PSI via distinct interactions compared to previously known PSI_3_-IsiA_18_ complexes. Time-resolved fluorescence (TRF) spectroscopy showed tight energetic coupling of IsiA to PSI, which serves a role in lowering antenna fluorescence yield and increasing the efficiency of energy transfer.

## RESULTS

### Overall structure of the PSI-IsiA supercomplex

Micrographs of the PSI-IsiA supercomplex [molecular weight (MW) ∼242 kDa, figs. S1 and S3] obtained with a Titan Krios (FEI) electron microscope were analyzed and processed with CryoSPARC using C1 symmetry, yielding a final resolution of 2.22 Å (fig. S1) (*45*). The consensus map was further locally refined to enhance resolution for specific volumes in the map and combined to form a composite map, which was used for model building and refinement (see Materials and Methods and fig. S2).

**Fig. 1.**
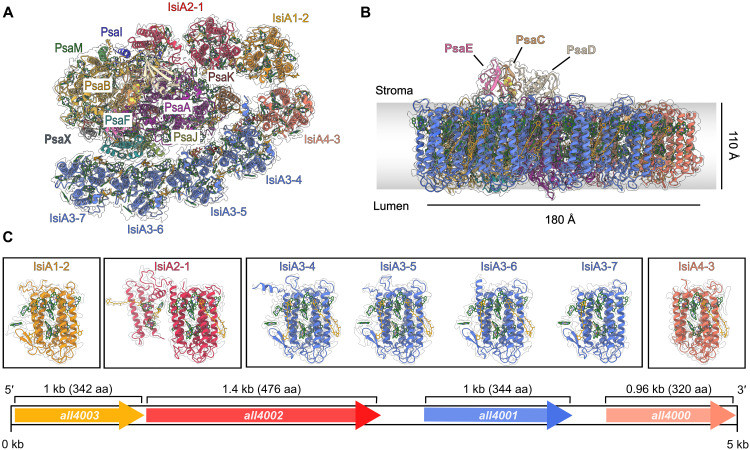
Specific IsiA subunits associate with the PSI core in *Anabaena* sp. PCC 7120. (**A**) Stromal view of the overall PSI-IsiA complex, which is composed of a monomeric core containing eight PSI subunits surrounded by heterogeneous PsaL-associated IsiAs (three subunits, red, orange and salmon) and homogeneous IsiA belt of four IsiA subunits (labeled by position, in blue). (**B**) Side view of the supercomplex from membrane plane. (**C**) Individual IsiA subunits are depicted with their cofactors and corresponding density and labeled according to their order in the genomic loci. The different gene products that were modeled are highlighted by color with *all4000* (salmon), *all4001* (royal blue), *all4002* (red), and *all4003* (orange).

The atomic model consists of a monomeric PSI with 11 polypeptide subunits surrounded by seven antenna subunits identified as products of IsiA genes (Fig. 1, A and B). The PSI monomer in our model is similar to previously published work, but the improved resolution allowed for the identification of the PsaK subunit sequence (Fig. 1A) (*36*). The PSI core coordinates 92 ChlA, 18 β-carotene (BCR), 3 echinenone, 2 phylloquinone, 3 [4Fe-4S] clusters, 3 2-dipalmitoyl-phosphatidyl-glycerole (LHG), and 2 1,2-distearoyl-monogalactosyl-diglyceride (LMG) molecules. In addition, the current resolution allowed for unambiguous assignment of all IsiA subunits to their respective genes. Four of the seven IsiA subunits were identified as products of the *all4001* gene and resemble the single IsiA gene found in many cyanobacteria, such as 6803 (Fig. 1C) (*42*, *43*).

The IsiA-PsaL fusion protein, gene *all4002*, was modeled as IsiA2-1. The IsiA belt in the structure coordinates 113 ChlA, 26 carotenes, 4 LHG, and 2 LMG molecules. High-performance liquid chromatography (HPLC) analysis suggested the presence of these pigments in addition to a number of oxygenated carotenoids, one of which (OH-BCR) was modeled as lutein (fig. S4). In total, 45 ChlA and 18 BCR molecules in addition to a few lipids in the current model are absent from the one previously available (fig. S5).

### Pigment interfaces and energy transfer in the PSI-IsiA supercomplex

**Fig. 2.**
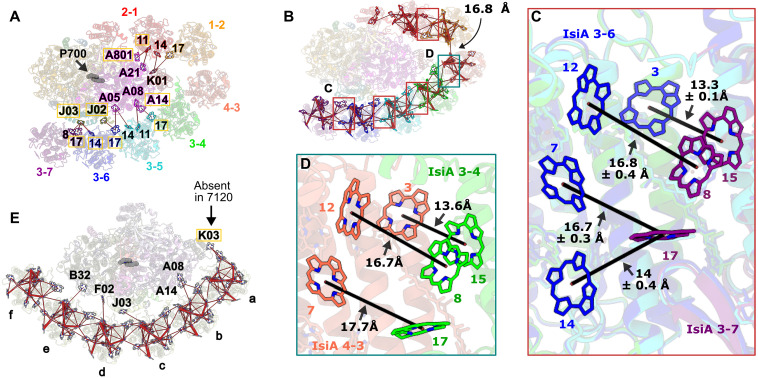
Energy transfer in the PSI-IsiA supercomplex of 7120. (**A**) Stromal view of the PSI-IsiA supercomplex showing ChlA pair FRET rates (red lines) between IsiA and PSI. High-efficiency energy transfer occurs differentially across PSI-IsiA interfaces and involves the PsaA/J/K subunits of the PSI core. (**B**) Corresponding FRET rates for ChlA network connections in the IsiA_7_ belt. (**C** and **D**) Close-up view of pigment connections at the IsiA-IsiA interfaces highlighted in (B) showing closest distances between central magnesium atoms (<18 Å). The four interfaces highlighted (in red boxes) in (B) are superposed in (C) with SD in ChlA Mg-Mg distances indicated to reflect the variance among them. (**E**) FRET rates in the PSI_3_-IsiA_18_ complex in 6803 (only one-third shown) suggest rerouting of main energy transfer pathway between IsiA and PSI from PsaK to PsaJ in 7120. The thickness of the red lines (FRET rates) is proportional to the magnitude of the energy transfer rate and its length to the distance between central Mg atoms. Only FRET rates within 12.5 to 0.15 ps lifetime range are shown, with intra-PSI rates not depicted for visualization purposes. Yellow outlines around ChlA identifiers signify chlorophyll that are <15 Å in proximity (Mg-Mg distance) in a pair between PSI and IsiA.

The PSI-IsiA supercomplex is a pigment-protein complex that participates in the harvesting and channeling of light energy into the reaction center pigments, P700, where charge separation takes place. Förster resonance energy transfer (FRET) rates and orientation factors were calculated for the homogeneous ChlA pigment array of 160 pigments to identify transfer pathways and terminal emitter sites (Fig. 2, A and B).

This analysis enabled us to identify the main channels of energy transfer from IsiA to PSI (Fig. 2A). The highest density of close pigment-pigment connections between PSI and IsiA occurs at the PsaJ and PsaL poles of PSI (Fig. 2A).

Notably, ChlA 517 (*17*) of the IsiA3-7 subunit forms among the closest interpigment connection observed between PSI and any of its antenna with a Mg-Mg distance of 11 Å to ChlA J03 (Fig. 2A). In addition, one of the terminal acceptors from IsiA in 6803 (K03) is absent in the 7120 PSI-IsiA structure, which instead reroutes energy transfer from IsiA through stronger couplings at the J subunit (Fig. 2, A and E).

Energy transfer rates between IsiA monomers are generally faster than the IsiA-PSI transfer (Fig. 2B). Close IsiA-IsiA pigment connections occur in four highly similar interfaces through the same ChlA clusters—3/7/12/14 and 8/15/17 (see Fig. 2C)—with small deviation in Mg-Mg distances, showing conserved coupling of pigments between the IsiA subunits. The IsiA4-3 and IsiA3-4 ChlA interface is unusual as it is missing ChlA 14 and the separation of the ChlA 17-7 pair is larger (Fig. 2D). Furthermore, when it comes to the interface of IsiA1-2 and IsiA4-3, only one major connection was observed with a Mg-Mg distance of 16.8 Å, suggesting a weak coupling between this pair and slower transfer rates between these two subunits (Fig. 2B).

### PSI-IsiA protein-protein interactions

**Fig. 3.**
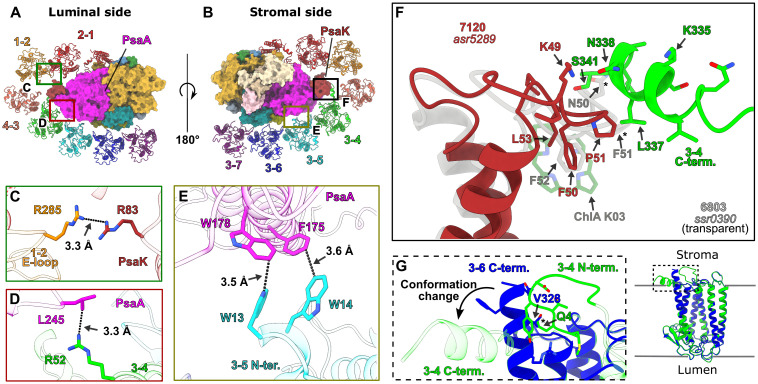
Key protein interactions and remodeling in the PSI-IsiA supercomplex of 7120. (**A**) Luminal view of the PSI-IsiA supercomplex from 7120 highlighting specific areas of interactions between PSI and IsiA. (**B**) Corresponding stromal view. (**C** to **E**) Close-up view of the PSI-IsiA protein-protein interaction surfaces highlighted in (A) and (B). (**F**) The PsaK protein (asr5289) in the iron stress–induced PSI-IsiA of 7120 accommodates interactions with the IsiA C terminus compared to the 6803 PsaK (ssr0390), which contains a patch of bulky phenylalanine residues (F51 & F52) that clash with it. (**G**) The IsiA3 subunits in positions 4 and 6 are superimposed, highlighting the incompatibility of the two different conformations that are characterized by a change in C-terminal orientation. The asterisk (*) denotes clashing between residues.

PSI and IsiA interact through numerous hydrophobic, ionic, and hydrogen bonding forces that exist at the protein-protein interface (Fig. 3). Using the contacts function of ChimeraX (overlap cutoff = 0.6), close protein-protein associations were identified on both stromal and luminal sides (Fig. 3, A and B). The PsaL N-terminal loop is fused to an IsiA domain forming the IsiA2-1 subunit. This region remains largely unperturbed and participates in extensive interactions with the PsaA, PsaD, and the PsaB subunits of PSI (fig. S6). Close contacts on the luminal side are scarce and include a unique arginine-arginine contact between PsaK and the E-loop of IsiA1-2 (Fig. 3C). Arginine residues have been observed to occur at protein surfaces and such like ion pairs and clusters may have a role in stabilizing higher-order oligomers (*46*–*48*). On the stromal side, IsiA3-5 N terminus and IsiA3-4 C terminus interact with PsaA and PsaK, respectively, through π−π aromatic interactions and electrostatic associations (Fig. 3, E and F).

### A unique PsaK subunit present in PSI-IsiA from 7120

The 7120 genome has three PsaK-like genes, one of which (gene ID: *asr4775*) has been previously modeled into the tetrameric PSI structure (Fig. 3F and fig. S7) (*35*). Homology models of all three sequences were docked into the PsaK density of our map and the proper sequence was chosen based on the best fit of the sequence side-chain densities (fig. S8). The corresponding PsaK gene (*asr5289*) lacks a short stretch of amino acids—FFGG—which coordinates ChlA K03. This pocket of residues is present in other PsaK sequences, and its absence results in the loss of the K03 pigment (fig. S7). Furthermore, the presence of this unique PsaK (*asr5289*) seems to be essential for the assembly of PSI-IsiA as the other forms of PsaK are expected to clash with the C terminus of the adjacent IsiA3 subunit (Fig. 3F).

Superposing one-third of the 6803 PSI_3_-IsiA_18_ complex onto the 7120 one shows differences in the C-terminal fold and interaction pattern of IsiA with PSI. In the PSI_3_-IsiA_18_ complex, the C terminus of IsiA is well folded and participates in specific interactions with PSI subunits PsaF/J and becomes increasingly disordered/flexible around PsaK (fig. S9A) (*42*, *43*), while in 7120, the opposite trend is observed where the IsiA3-4 C terminus is most ordered and participates in electrostatic interactions with PsaK, while becoming disordered around the PsaJ/F junction (fig. S9A). In particular, one lysine residue (K49) in the PsaK of 7120 and absent from the PsaK of 6803 is involved in specific interactions with the C terminus of IsiA3-4, further highlighting the importance of this specific PsaK subunit in the PSI_1_-IsiA_7_ structure (Fig. 3F and fig. S7). Thus, while IsiA seems to be occupying similar positions in the two complexes, the mode of interaction between PSI and IsiA is quite different between PSI_3_-IsiA_18_ and PSI_1_-IsiA_7_.

### Structural differences among IsiA subunits

The PSI_1_-IsiA_7_ complex from 7120 contains different IsiA gene products that are associated with the PSI core (Fig. 1, A and C). The most abundant IsiA gene product in our model (IsiA3) coordinates a total of 17 ChlA and 4 BCR except for IsiA3-7, which contains only 2 BCR. One reason for this could be the lower local resolution of this subunit compared to the ones adjacent to it (fig. S2).

The main difference between the four IsiA3 proteins associated with PSI is a unique C-terminal conformation of IsiA3-6, which folds upward instead of toward PSI as in IsiA3-4 (Fig. 3G). Alignment of all IsiA3 subunits shows that the upward position of the C terminus observed in IsiA3-6 is incompatible with the position of the N terminus observed in other IsiA3 subunits, suggesting that the upward conformation of the C terminus is an alternative conformation of IsiA3, and involved a different (possibly disordered) conformation of the IsiA3 N terminus (Fig. 3G).

**Fig. 4.**
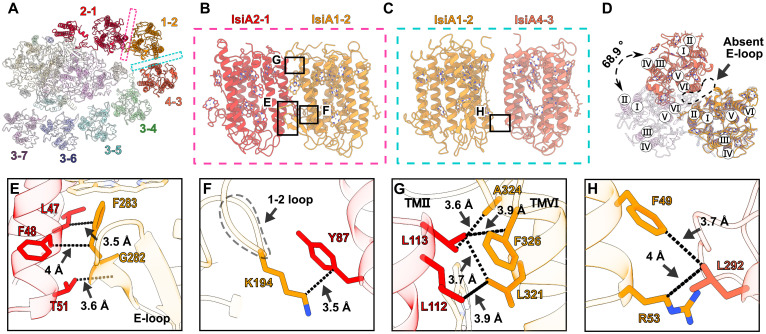
IsiA-IsiA interaction interfaces in the 7120 PSI-IsiA supercomplex. (**A**) Overview of the PSI-IsiA supercomplex in 7120 with interfaces formed by the three subunits IsiA 2-1/1-2/3-2 highlighted. (**B** and **C**) Side view of IsiA dimers that interact through protein-protein patches between adjacent subunits. (**D**) Alignment of IsiA-IsiA dimers reveals a ∼70° rotation in the 1-2/4-3 dimer relative to the others with the absence of E-loop motif important for association of adjacent subunits. (**E** to **G**) Close-up view of different binding patches in the IsiA2-1 and IsiA1-2 dimer and the protein residues/motifs that hold it together. (**H**) The IsiA1-2 and IsiA4-3 interface involves very few amino acid interactions and a large gap between the dimers.

Two other gene products were modeled—IsiA1-2 and IsiA4-3. IsiA1-2 lacks 2 ChlA compared to IsiA3 and contains a discernably different E-loop sequence that coordinates ChlA 517, which interacts with PSI and a non–α-helical C terminus (figs. S11 and S12). The C terminus of IsiA1-2 does not interact with PSI but loops backward instead (fig. S11). This is different from other IsiA structures where the C terminus has been seen to be highly flexible and facilitate interactions with PSI at the PsaF/J junction (*43*). The smallest IsiA subunit, IsiA4-3, lacks 4 ChlA and does not contain an E-loop (fig. S12). The lack of the E-loop results in a weakly associated IsiA dimer between the IsiA1-2 and IsiA4-3 pair, which occurs at the elbow of the IsiA belt (Fig. 4D).

### IsiA-IsiA association interfaces

There are four unique interfaces between IsiA subunits in terms of protein-protein interactions (Fig. 4 and fig. S10). Interactions are mostly hydrophobic in nature and occur in both stromal and luminal regions (Fig. 4B and fig. S10). While the protein-protein interaction residues and patches remain largely unchanged in the 7120 supercomplex, two unique interfaces are present in this complex involving the three identified subunits: IsiA2-1, IsiA1-2, and IsiA4-3 (Fig. 4A). A small extension loop on the IsiA1-2 side facing away from PSI (1-2 loop) that is absent from other IsiA contains K194 that interacts with IsiA2-1 tyrosine (Y87) (Fig. 4F). In nearly all IsiA dimeric interfaces, the stromal halves of TMH II and VI of adjacent monomers provide additional hydrophobic packing between them (Fig. 4G and fig. S10).

**Fig. 5.**
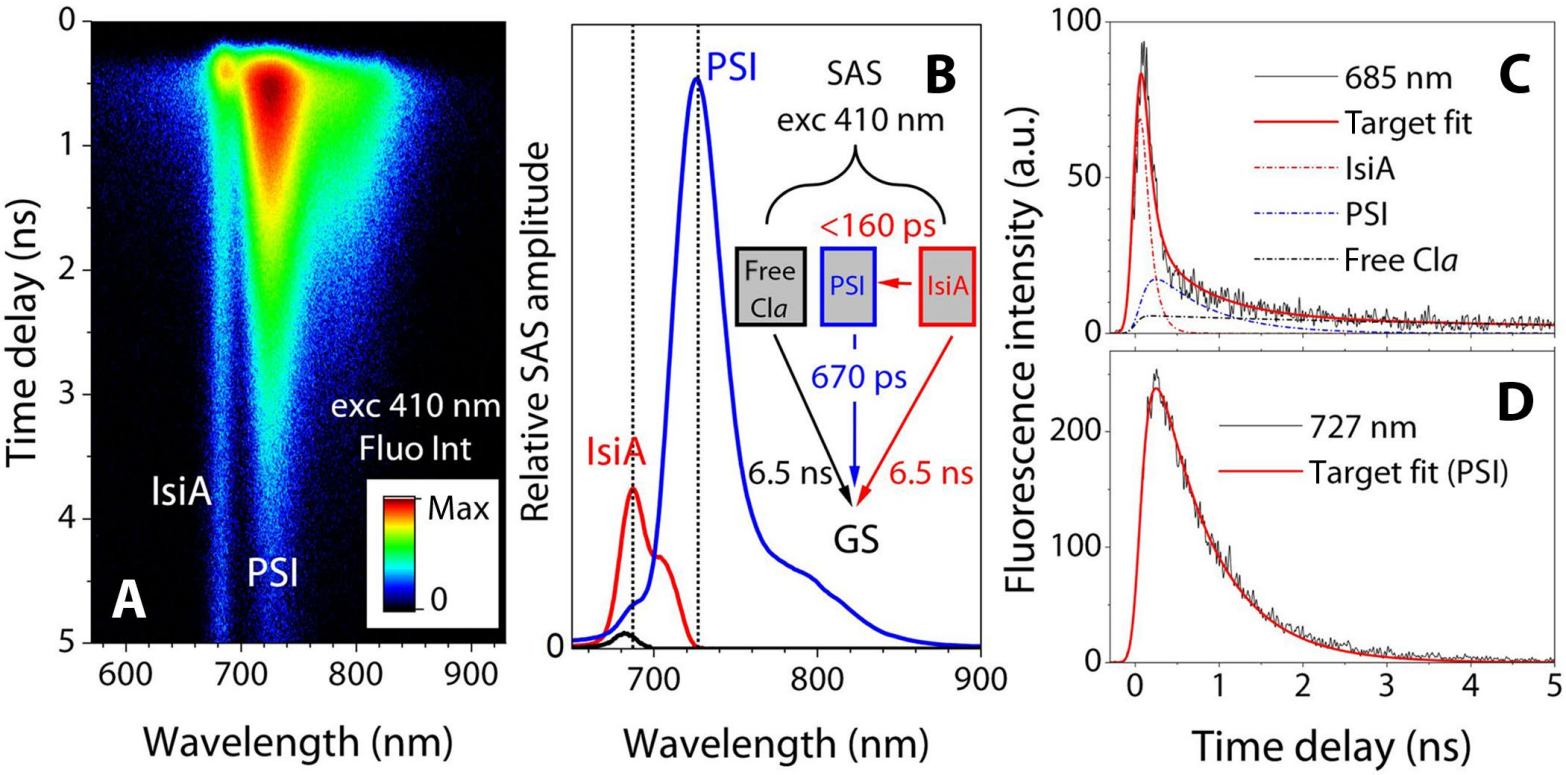
Time-resolved fluorescence of the IsiA-PSI supercomplex at 77 K. (**A**) Pseudo-color fluorescence decay map recorded after excitation at 410 nm with fs laser. Two main fluorescence bands associated with IsiA and PSI as indicated in the panel. For enhanced visibility of weakly fluorescing components, the color scale was plotted in log. (**B**) Target analysis of fluorescence decay map from (A) according to the kinetic scheme sketched in the panel. (**C** and **D**) Representative fluorescence decay traces extracted from the fluorescence decay map at wavelengths corresponding to maximum of IsiA (C) and PSI (D) fluorescence. Both traces are accompanied with fitted traces that, if needed, were decomposed to time-dependent contribution of principal fluorescing components; SAS, species-associated spectrum; GS, ground state.

The interactions between adjacent IsiA3 are almost entirely between hydrophobic amino acids with the possibility of a hydrogen between K279 and serine (S51) (fig. S10A). In the case of the IsiA4-3/3-4 dimer, the well-folded C terminus of IsiA3-4 also interacts with IsiA4-3 (fig. S10B). The IsiA1-2 and IsiA4-3 dimer interface contains only two close contacts, suggesting weak binding of those two subunits (Fig. 4H). The absence of an E-loop, an important structural element in IsiA-IsiA association, in IsiA4-3 alters its interaction and orientation (rotated by 68.9°) with respect to IsiA1-2 compared to the other dimers (Fig. 5D). The E-loop of each IsiA subunit interacts with the adjacent monomer extensively through hydrophobic forces (Fig. 4E and fig. S10).

### Spectroscopy of the PSI-IsiA supercomplex

Absorption and fluorescence emission spectra of IsiA-PSI recorded at room temperature and at 77 K are given in fig. S13C. Absorption spectrum consists of typical features associated with absorption of pigments found in those complexes. At room temperature, ChlA shows Soret and Qy bands at 436 and 673 nm, respectively. The carotenoid (BCR) band, associated with electronic transition to the second excited singlet state of the pigment, appears in the 480- to 500-nm range with the (0-0) vibronic band at 493 nm. Fluorescence emission at room temperature appears at 681 nm with vibronic overtone at ∼730 nm. Considering that at room temperature fluorescence emission from PSI is expected to be very weak and spectrally should resolve broad featureless band with maximum located ∼700 nm (*49*), the observed emission spectrum of IsiA-PSI is mostly associated with emission from the antenna complex.

Lowering temperature from 293 to 77 K leads to noticeable spectroscopic changes. As seen, least affected is the ChlA Soret band. On the other hand, vibronic peaks of β-carotene absorption gain fine resolution and shift to longer wavelengths by 5 nm. In the ChlA Qy range, the most apparent change is the formation of a long-wavelength band at 708 nm, which is associated with a red ChlA, a characteristic for PSI from cyanobacteria (*50*). The formation of ChlA red traps has a profound effect on fluorescence and triggers the onset of a long-wavelength fluorescence band that, for the 7120 PSI, has a maximum at 726 nm. Emission from ChlAs in IsiA is still visible through the band and is bathochromically shifted to 685 nm. At 77 K, the IsiA band amplitude is only ∼20% of the PSI band, indicating that the IsiA subunits and PSI are strongly coupled and fluorescence from IsiA is greatly quenched by very efficient IsiA-to-PSI excitation energy transfer. This subject was further investigated at 77 K with the application of TRF. The results are shown in (Fig. 5).

Figure 5A presents a pseudo-color fluorescence decay map recorded after excitation at 410 nm. Assignment of the two main fluorescence bands is indicated in the panel. Note that to enhance the visibility of weakly fluorescing species, a color scale was plotted in log scale; therefore, intensities of dominant bands will be much flatter compared to comparable presentation in a linear scale. The map reveals two dominant bands. One is associated with fluorescence of bulk of IsiA and very quickly fades away within 300 ps after excitation. The second is, as expected, associated with PSI (see notes on the map) and consists of the majority of the observed fluorescence signal. The map also reveals much weaker long-lived fluorescence band localized at ∼680 nm. The dynamic properties of the fluorescing species were obtained by performing target analysis of the fluorescence decay map. The results of the fitting and scheme of the fitting model are provided in Fig. 5B. Because excitation at 410 nm is not preferential and will excite ChlA in all three fluorescent species, it was assumed that excitation simultaneously populates all of them. Subsequently, bulk of IsiA transfers excitation to PSI with a time constant that seems to be faster than temporal resolution of the setup in this hardware configuration (∼160 ps), and this transfer rate is extremely competitive to the intrinsic decay of ChlA in stand-alone IsiA that, based on previous investigation, is ∼7 ns at 77 K (*51*). The long-lived fluorescence component seems to be completely decoupled from PSI as characterized by fluorescence decay lifetime typical for the intrinsic decay of excited singlet state of monomeric ChlA. This emission is associated with either free-floating ChlA (impurities) or some remnant fraction of uncoupled IsiA. The panel shows spectro-kinetic results of target analysis, SAS—species-associated spectra. Their relative amplitudes indicate their maximal relative contributions in the TRF map. Two final panels, Fig. 5 (C and D), show exemplary fluorescence decay traces extracted from the TRF map. Those are taken for characteristic wavelengths near the fluorescence maxima of PSI and PSI-coupled IsiA as indicated by vertical lines in Fig. 5B. The raw traces are accompanied by global fits, which, if necessary, were decomposed into time-dependent contributions of participating fluorescence components. Overall, these results show that the studied sample consists of a very pure IsiA-PSI supercomplex with IsiA antenna tightly coupled to PSI as indicated by the efficient excitation energy transfer process that strongly quenches IsiA fluorescence emission.

## DISCUSSION

PSI is present as a tetramer in 7120 in contrast to many other cyanobacterial species that contain a trimeric PSI. In iron stress, these organisms express a pigment-protein antenna called IsiA that is associated with PSI and assists in light harvesting and energy transfer. In this work, we have provided details on the structure of the PSI_1_-IsiA_7_ antenna complex from 7120 that were previously unseen including a substantial number of cofactors (fig. S5). The most noteworthy aspect of our model is the identification and structural analysis of all different IsiA gene products in terms of their association with PSI and each other. The IsiA hetero-oligomer (near the PsaL pole) composed of the three identified IsiA proteins (IsiA2-1, IsiA1-2, and IsiA4-3) and the IsiA3 homo-oligomer form a belt around the PSI core (Fig. 1A). Our data show the presence of an additional seventh IsiA3 subunit, bringing the count of IsiA3 to four in the homo-oligomer. This seventh IsiA3 is a good candidate for being a terminal emitter from IsiA3’s to PSI because its ChlA 17 is within 11 Å of a PSI ChlA, one of the shortest distances known between PSI and its antenna (*43*, *52*, *53*). In addition to this, four other ChlA pairs between PSI and IsiAs are within ∼15 Å (Fig. 2A). This large number of closely situated pigments compared to PSI_3_-IsiA_18_ complexes contributes to the high efficiency of antenna-core energetic coupling as indicated by time-resolved spectroscopy measurements (Fig. 5 and table S2). Unlike other PSI-IsiA, the one in 7120 involves a monomeric core, and this arrangement may necessitate multiple close antenna connections to compensate for slower IsiA-IsiA transfer times compared to PSI_3_-IsiA_18_ systems. The absence of an E-loop, a major IsiA-IsiA interaction motif, in IsiA4-3 hinders close association with the adjacent IsiA2-1 (Fig. 4, D and H). In addition, the lack of close pigment pairs between IsiA4-3 and IsiA2-1 suggests a discontinuity in the energetic connectivity of the IsiA belt at this site (Fig. 2B). It is possible that IsiA4, as opposed to other E-loop containing IsiA, provides the necessary dimeric interface that can accommodate the asymmetric photosystem-antenna arrangement of the PSI_1_-IsiA_7_ complex. Changes of such nature in pigment protein complexes occur through concerted mutation and evolution of proteins that remodel antenna photosystems. One way this is observed in the current structure is through the PsaK (asr5289) subunit, which does not contain the K03 binding pocket but instead seems to have an amino acid interface suitable for specific interactions with the IsiA3-4 C terminus (Fig. 3F). PsaK (asr5289) in our structure is one of three PsaK genes found in the 7120 genome (fig. S7D), and its expression may be tied to iron starvation. PsaK (asr5289) directly interacts with IsiA3-4, distinguishing this complex from PSI-IsiA in other cyanobacteria, where specific interactions are concentrated at the PsaF/J region of the core (fig. S14). This difference is unusual given that the PSI core in all these systems align almost perfectly and raises questions as to what exactly regulates the association of the IsiA antenna to PSI (fig. S14). The lower negative potential of IsiA3 and its C terminus in 7120 compared to other IsiA may reflect weak electrostatic binding to PsaF/J and explain the high disorder of the C terminus in this region (figs. S9A and S14) (*54*).

Some of these differences in structure are not readily predictable from sequence analyses. One such observation is the relative distances between the stromal aromatic residues interfacing PsaA and the N terminus of IsiA3-5, which are much closer in this model compared to PSI-IsiA from 6803 (fig. S9B). On the other hand, the conformation of the E-loop (luminal side) in 6803 seems to favor interactions with PSI. One of the subunits facing PsaF/J, IsiA3-6, displays an upward fold of its C terminus, which clashes with the ordered N-terminal conformation observed in other IsiA positions (Fig. 3G). One possibility is that the flexible C terminus of IsiA at this location could serve as a binding location for other proteins such as phycobilisomes and important for state transitions (*55*). Another member of the PCB/IsiA family, called IsiX, contains a C-terminal extension of 127 residues and has been suggested to play a role in interacting with far-red allophycocyanin (*56*).

**Fig. 6.**
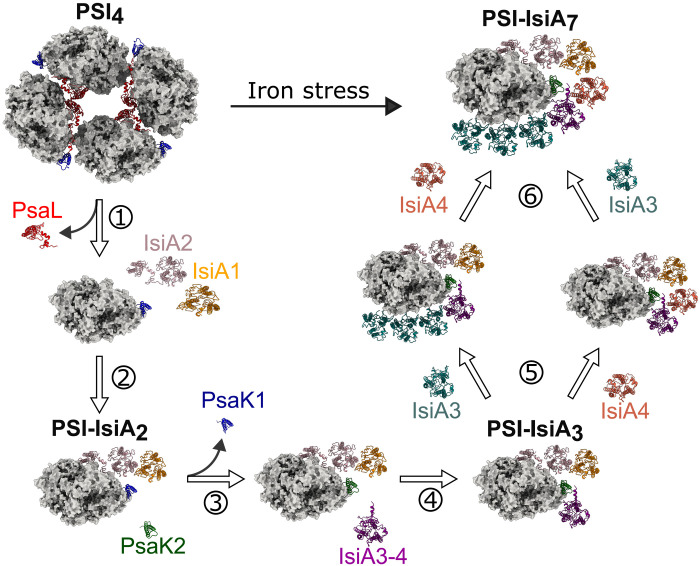
Mechanism of PSI-IsiA assembly in 7120. The process is initiated with a population of tetrameric PSI (with PsaL and PsaK1) under iron-replete conditions that undergo monomerization (1) upon iron stress that also triggers expression of all IsiA genes. This is followed by the attachment of the IsiA2 protein that contains the PsaL fusion and subsequently its partner IsiA1 forming the PSI-IsiA_2_ complex (2). At this stage, PsaK2 (asr5289) replaces PsaK1 (asr4775), allowing IsiA3 to bind through its C terminus forming the PSI-IsiA_3_ complex (3 and 4). Only after this can IsiA4 and other IsiA3 bind and complete the transition from a tetrameric PSI to a PSI-IsiA_7_ assembly (5 and 6).

On the basis of our structure, we have proposed a scheme for the assembly and formation of PSI-IsiA in 7120 (Fig. 6). In contrast to the ChlA-ChlA couplings and transfer of energy between IsiA and PSI, which are localized on the PsaJ/L poles of PSI, the assembly mechanism is driven by protein-protein interactions, which are strongest at the PsaK interface. Iron stress induces the expression of IsiA antenna, which crowds thylakoid membranes, beginning the transition from tetrameric PSI cores to PSI-IsiA complexes. As suggested previously, the first committed step after monomerization is likely the attachment of IsiA2-1 with its PsaL domain onto PSI (*36*). At this stage, it is also possible for IsiA1-2 to attach forming the PSI-IsiA_2_ complex. On the basis of the presence of a different PsaK isoform that participates in the strongest interactions with IsiA3 (Fig. 3F) in our structure, we think that the presence of this subunit is crucial for complete formation of PSI-IsiA_7_. Upon the binding of IsiA3-4 to PsaK2 (asr5289), IsiA oligomerization can begin on the PsaF/J side of PSI extending the belt. Because of its stronger interactions with IsiA3, it is possible that IsiA4 binds after the association of its partner, IsiA3-4, to PSI. The sucrose gradient fractionation of thylakoid membrane pigment protein complexes from 7120 in iron stress showed an additional lower–MW green band (fig. S13, A and B). The corresponding SDS–polyacrylamide gel electrophoresis analysis indicates a lower enrichment of IsiA1/3/4 in this ChlA complex, which could be an intermediate monomeric PSI-IsiA2–like complex. Nevertheless, the genetic identity of the PsaK in this complex needs to be experimentally verified to offer concrete evidence for a PsaK-dependent oligomerization mechanism. Physiologically, it is not yet clear if these PSI-IsiA states represent different stages of the iron stress response and would require profiling the expression of different IsiA genes in the course of iron stress and fractionation of thylakoid membranes at different iron concentration regimes.

Overall, our findings highlight the diversity that exists in PSI antenna and how genetic remodeling shapes the photosynthetic machinery to adapt to environmental pressures. In addition, our data enabled us to look at the PSI-IsiA assembly from 7120 in detail, providing the basis for future theoretical, genetics, and spectroscopic studies on this complex.

## MATERIALS AND METHODS

### Cell growth

Cultures of 7120 were grown photoautotrophically in glass bottles in 10-liter batches in liquid BGII media at 30°C with bubbling. Media was supplemented with 12 ng ml^−1^ ferric ammonium citrate for the induction of iron stress and expression of IsiA. The growth light intensity used was 25 μmol photons m^−2^ s^−1^ (LI-250A LI-COR sensor) supplied from a light-emitting diode array (Fluence RAY).

### Purification of the PSI-IsiA complex

Cells were grown for 3 weeks before harvesting by centrifugation (12,000 rpm, 3 min) in an F9-6 × 1000 LEX rotor using a Sorval LYNX 6000 centrifuge (Thermo Fisher Scientific). Pellets were suspended in STN1 buffer (30 mM Tricine-NaOH, pH 8, 400 mM sucrose, and 15 mM NaCl) and lysed with three cycles at 206.8 MPa in a cell disruptor (Constant Systems Ltd). The resulting lysate was centrifuged (12,000 rpm, 5 min) in an F20-12 × 50 LEX rotor (Thermo Fisher Scientific) to remove insoluble debris before resuspending in STN1. Membranes were pelleted through ultracentrifugation (Ti70 rotor, 45,000 rpm, 2 hours, 4°C) and suspended in STN2 (30 mM Tricine-NaOH, pH 8, 400 mM sucrose, and 150 mM NaCl) buffer. The suspension was incubated on ice for 10 min and membranes were re-pelleted through ultracentrifugation (Ti70 rotor, 45,000 rpm, 2 hours, 4°C) and kept in STN1. Thylakoid membranes suspended in STN1 buffer were solubilized with *n*-dodecyl β-d-maltoside (DDM, Glycon) detergent (15:1 DDM:ChlA ratio) for 30 min on ice (inverting tube every 10 min). After spinning down (Ti70 rotor, 45,000 rpm, 30 min, 4°C), the dark green supernatant containing solubilized complexes was collected. The salt (NaCl) concentration was increased to 200 mM and the sample was precipitated with 10% polyethylene glycol, MW 3350 (Hampton Research). The mixture was centrifuged in a F20-12 × 50 LEX rotor (5000 rpm, 5 min) and the precipitate was suspended in 0.02% DM, 30 mM Tricine-NaOH, pH 8, and 75 mM NaCl and loaded onto a 10 to 30% (w/v) sucrose gradient (same buffer) for overnight ultracentrifugation (Beckman SW60 rotor, 36k rpm, 16 hours, 4°C). The highest MW green band corresponding to PSI-IsiA was collected for further analysis (fig. S13, A and B).

### Clear native gel

Clear native gel as shown in fig. S3 was performed based on the protocol detailed in (*57*).

### Pigment analysis

HPLC was used to profile pigments in the PSI-IsiA supercomplex of 7120 according to the protocol in (*58*). To extract pigments, the largest– MW green band (fig. S13A) was incubated at room temperature for 5 min in 80% acetone. After centrifugation in an Eppendorf 5424 R benchtop centrifuge (14,000 rpm, 10 min), 60 μl of the upper hydrophobic layer was injected into the HPLC apparatus (Agilent Technologies 1100 series HPLC) with an Agilent ZORBAX Eclipse Plus C18 column (4.6 mm × 100 mm, 3.5 μm) calibrated to 20°C fitted with a G1322A degasser, a G1311A quaternary pump, a G1313A ALS autosampler, a G1314A VWD detector, and a G1364A AFC automatic fraction collector. The detection wavelength and flow rate were set to 440 nm and 2 ml min^−1^, respectively. Equilibration was achieved using mobile phase A (82:18 acetone:H_2_O) before injection. For the actual run, mobile phase A was pumped for the first 2 min, followed by mobile phase B (7:0.96:0.04:2 acetonitrile:MeOH:H_2_O:ethyl acetate) for 1 min, and lastly mobile phase C (7:0.96:0.04:8 acetonitrile:MeOH:H_2_O:ethyl acetate) until the end of run at 8 min postinjection.

### Sample preparation for single-particle cryo-EM analysis

The green PSI-IsiA band collected from the sucrose gradient was buffer exchanged to 30 mM Tricine-NaOH, pH 8, 75 mM NaCl, and 0.02% DDM using a Sephadex G-50 gel filtration column. The sample was concentrated using spin columns (Spin-X UF 100k, Corning) to a ChlA concentration of 1.0 mg ml^−1^. Three microliters of this sample was applied to a holey carbon grid (C-flat 1.2/1.3 Cu 300 mesh, Protochips) presoaked in buffer, and vitrification was accomplished through flash-plunging into liquid ethane using an automated Vitrobot Mark IV (Thermo Fisher Scientific/FEI) with a blotting time of 3 s (humidity of 100%, blotting force −1). Grids were stored in liquid nitrogen before screening and data acquisition.

### Data acquisition

Cryo-EM imaging was done using a Titan Krios transmission electron microscope (Thermo Fisher Scientific/FEI) at an acceleration voltage of 300 keV. Images were recorded on a K3 (Gatan Inc.) equipped with a SelectrisX energy filter with a slit width of 20 eV. Magnification was set to 165,000×, resulting in a pixel size of 0.731 Å/pixel. The targeted defocus range was varied from −0.8 to −2.5 μm using the EPU software (Thermo Fisher Scientific).

### Data processing

A total of 6169 movies were imported and processed using cryoSPARC (*45*). Movies were first motion corrected with dose weighting using “Patch motion correction” and then defocus parameters were estimated with “Patch CTF estimation.” Blob picker was used to pick 975,718 particles, which were then inspected, extracted (box size of 810 pixels), and Fourier cropped to 128 pixels. This set of particles was subjected to three rounds of two-dimensional (2D) classification before ab initio maps were calculated (two classes) to clean the dataset (*45*). Particles images corresponding to the major class (101,156) were then re-extracted (Fourier cropping set to 540 px), 2D classified, and then subjected ab initio reconstruction (*45*). The ab initio map was then used to perform nonuniform refinement with both defocus and Global CTF refinement switched on to generate the consensus map at a resolution of 2.2 Å (*59*). The consensus map was further processed to produce three focused maps, which were combined into a composite map using the “combine_focused_maps” program available in the Phenix suite (*60*). One of the three focused refinements was carried out by local refining the consensus map with a PSI core mask. The other two focused refinements were performed after a round of masked 3D classification (10 classes) with corresponding masks: IsiA_1–5_ and IsiA_6,7_. Mask bases were created with “molmap” in UCSF ChimeraX and imported into cryoSPARC to convert to masks using voltools (*45*, *61*). Final resolutions were estimated using the gold standard Fourier-shell correlation method at a cutoff of 0.143 (*62*). A detailed overview of the processing workflow is available in figs. S1 and S2.

### Model building and refinement

The previously published atomic model of PSI-IsiA (Protein Data Bank ID: 7Y3F) was used as a starting point for refinement and model building using our EM map (*36*). Initially, the model was rigid body docked into the composite map using the “fit in map” tool of UCSF ChimeraX software (*61*). Refinement of model was accomplished using the “real_space_refine” program from Phenix and manual refinement with Coot (*60*, *63*, *64*). ChlA and BCR were built into the different IsiA map regions using Coot. Homology models of the different IsiA genes and PsaK sequences were acquired using AlphaFold predictions, and their fit into previously unmodeled regions was assessed based on recognizable side-chain density (figs. S8 and S15) (*65*). Model validation statistics, cross-correlations, and *Q* scores are shown in tables S1 and S3.

### Sample handling and basic spectroscopic methods

Steady-state absorption spectra of the IsiA-PSI sample were recorded using a UV-Vis 1800 spectrophotometer (Shimadzu, Japan). For cryogenic measurements, sample was held in the liquid nitrogen vapor-based, temperature-controllable VNF-100 cryostat (Janis Research, USA). To obtain transparent glass at low temperature, glycerol was added to the sample to total concentration of 60% (v/v) prior measurements, and the sample was transferred to a 1-cm polymethyl methacrylate (PMMA) cuvette. Fluorescence emission at room temperature was obtained using a Shimadzu RF-6000 fluorometer (Shimadzu, Japan). The sample was excited at 440 nm, corresponding to the Soret band of ChlA. The bandwidths of excitation and emission slits were set to 5 nm. To minimize scattering effects, a 610-nm long-pass optical filter was placed on the detector entrance. Expected steady-state fluorescence emission at 77 K was obtained by time integration of TRF emission contours obtained from a streak camera setup (vide infra).

### Time-resolved fluorescence

A streak camera system based on a universal C5680 streak camera from Hamamatsu, coupled to spectrograph from Bruker, was used for TRF experiments and was described in detail previously (*49*). Briefly, the streak camera system was coupled to the ultrafast-laser system, consisting of an ultrafast Ti:Sapphire laser (Mai-Tai, Spectra-Physics, USA), an ultrafast optical parametric oscillator (Inspire100, Spectra-Physics, USA), and a pulse selector (Model 3980, Spectra-Physics, Milpitas, USA) used to lower the frequency of the excitation beam to 8 MHz. The excitation beam was depolarized before the sample using an achromatic depolarizer (DPU-25, Thorlabs Inc., USA). The excitation beam was set to 410 nm, focused on a circular spot of ∼1 mm diameter and set to a low photon density of ∼10^10^ photons/cm^2^ per pulse that warranted annihilation free excitation migration pigment array of the IsiA-PSI supercomplex. To avoid light scattering effects, a 610-nm long-pass filter was placed at the spectrograph entrance.

### Target analysis of TRF

The TRF dataset was fitted with application of target analysis. In general, TRF decay signals at any time delay and wavelength, Fl(*t*, λ), can be decomposed to a superposition of *n*th **C*_*i*_(*t*)*⋅*SAS_*i*_*(λ) (species-associated spectra) products (*66*): *Fl (t ,* λ*)* = $\sum_{i=1}^{n}$ **C*_*i*_(*t*)SAS_*i*_(*λ*)* where **C*_*i*_(*t*)* are time-dependent SAS concentrations, defined by a kinetic model intended to mimic the true excitation decay pathway and typically reflect complex interactions between mutually dependent fluorescing species. This modeling includes anticipated microscopic rates between interacting species and, unlike typical unbiased global fitting producing macroscopic (observed) transfer rates, focuses rather on correct decomposition of datasets to well-defined spectral components, or so-called molecular species. In such a model, the initially populated concentration(s) is/are convoluted by the excitation pulse represented by the instrument response function, IRF. Target analysis was performed using CarpetView (Light Conversion, Lithuania). The fitting included convolution of the data with Gaussian-like IRF having the full width at half maximum of ∼160 ps. All plots were done in Origin 2025 (Origin Lab Corp., USA).

### Förster rate calculations

Förster rates and dipole-dipole orientation factors were calculated similarly to (*43*, *53*). The Förster equation for calculating energy transfer rates from donor (D) to acceptor (A) used in this study was $K_{\text{DA}}=\frac{C_{\text{DA}}K^{2}}{N^{4}R_{\text{DA}}^{6}}$, where $C_{\text{DA}}$ represents the spectra overlap integral between the donor and acceptor pair. The values of the refractive index, *N* (=1.55), and the overlap integrals were taken from estimates in (*67*). $K^{2}$ is the dipole orientation factor given by ${\left[{\hat{\mu }}_{D}\cdot {\hat{\mu }}_{A}-3\left({\hat{\mu }}_{D}\cdot {\hat{R}}_{\text{DA}}\right)\left({\hat{\mu }}_{A}\cdot {\hat{R}}_{\text{DA}}\right)\right]}^{2}$ with ${\hat{\mu }}_{D}$ and ${\hat{\mu }}_{A}$ representing the unit dipole vectors of donor and acceptor pigments, respectively (vector defined by the $N_{B}$ and $N_{D}$ nitrogen atoms of each pigment). The distances between central magnesium atoms of each interacting pigment pair, $R_{\text{DA}}$, were obtained from the structural model. A python script adapted from (*43*) was used to compute parameters directly from the model.
