# Supplementary material for: Antenna photosystem I remodeling in the iron stress–induced complex from Anabaena sp. PCC 7120
Source: Sci Adv. 2026 Aug 12;12(33):eaeg3567. doi: 10.1126/sciadv.aeg3567 (PMC13464640; doi:10.1126/sciadv.aeg3567)
Supplement: Supplementary file 1 [file sciadv.aeg3567_sm.pdf]

Science Advances
AAAS

Supplementary Materials for

Antenna photosystem I remodeling in the iron stress–induced complex from *Anabaena* sp. PCC 7120

Ranel Maqdisi *et al.*

Corresponding author: Yuval Mazor, ymazor@asu.edu

Sci. Adv. **12**, eaeg3567 (2026)
DOI: 10.1126/sciadv.aeg3567

The PDF file includes:

Figs. S1 to S16
Tables S1 to S3
Legend for data S1

Other Supplementary Material for this manuscript includes the following:

Data S1

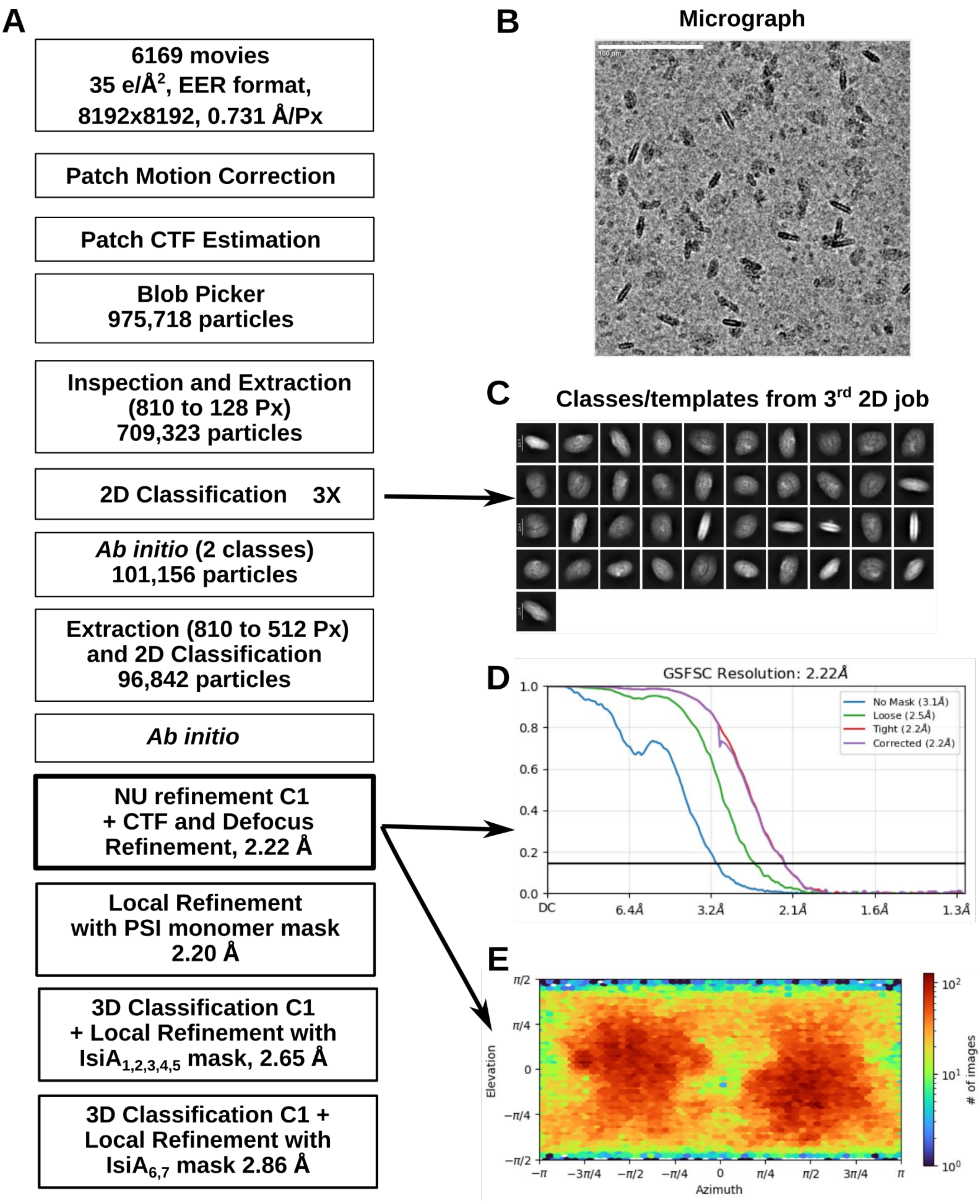

Figure S1 – Data Processing with cryoSPARC. A. General overview of processing workflow. **B**. Exemplary micrograph image of grids showing PSI-IsiA particles in different orientations. **C**. 2D classes of the particle which were used for initial *ab initio* job. **D**. Golden standard Fourier shell correlation plot for the NU refined consensus map. **E**. Corresponding angle distribution plot for refined poses. NU – Non-uniform refinement.

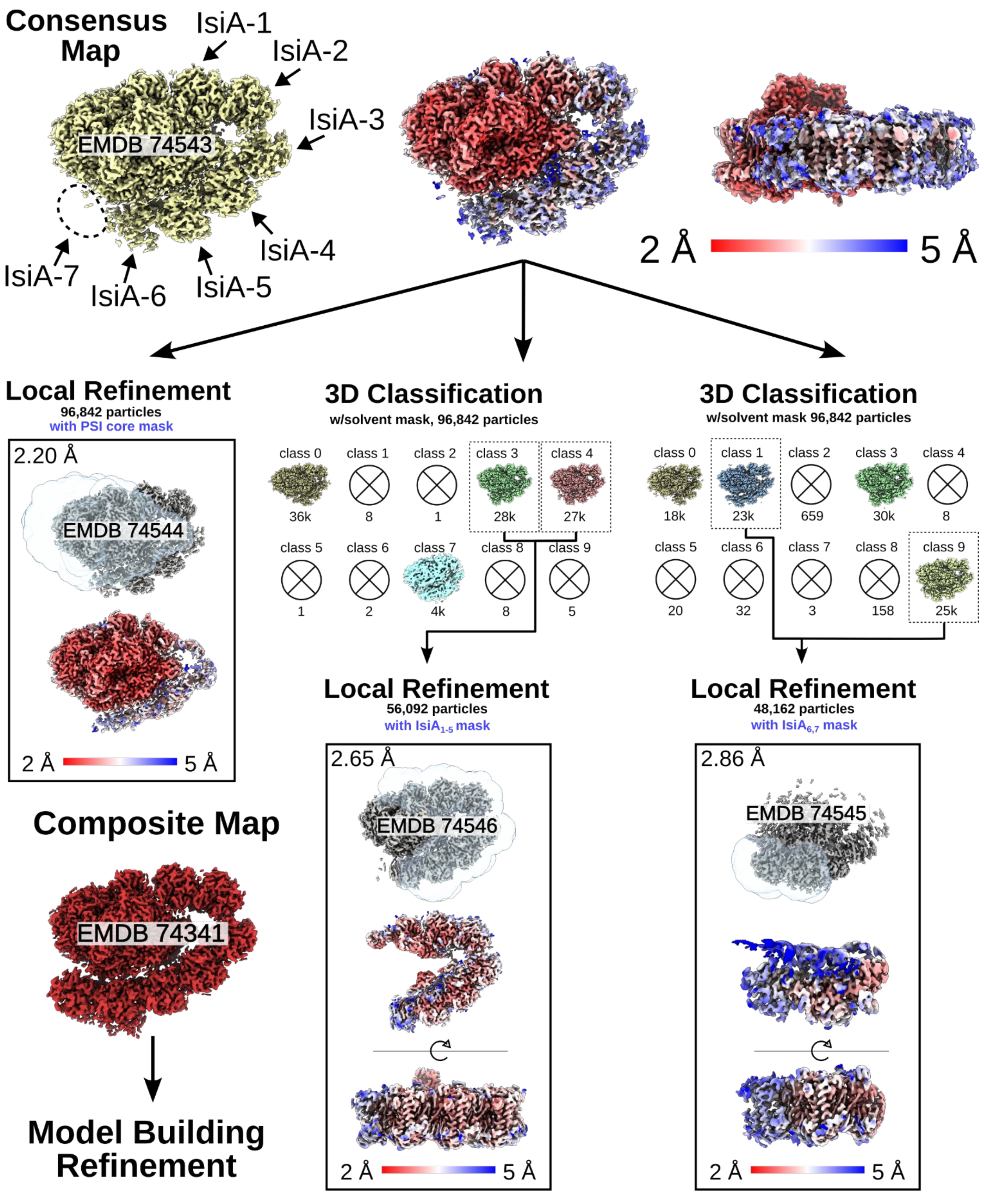

Figure S2 - Detailed overview of additional processing of consensus map (EMDB 74543). Processing was done with cryoSPARC and involved the production of three focused refined maps that were combined to form the final composite map (EMDB 74341) used for model building and refinement. The consensus map was locally refined with a PSI core mask to yield EMDB 74544. For the other two maps, 3D classification with solvent mask was performed and particles from select classes were used for local refinement to enhance resolution in different regions of the IsiA belt. Local resolution maps are shown for each of the locally refinement maps.

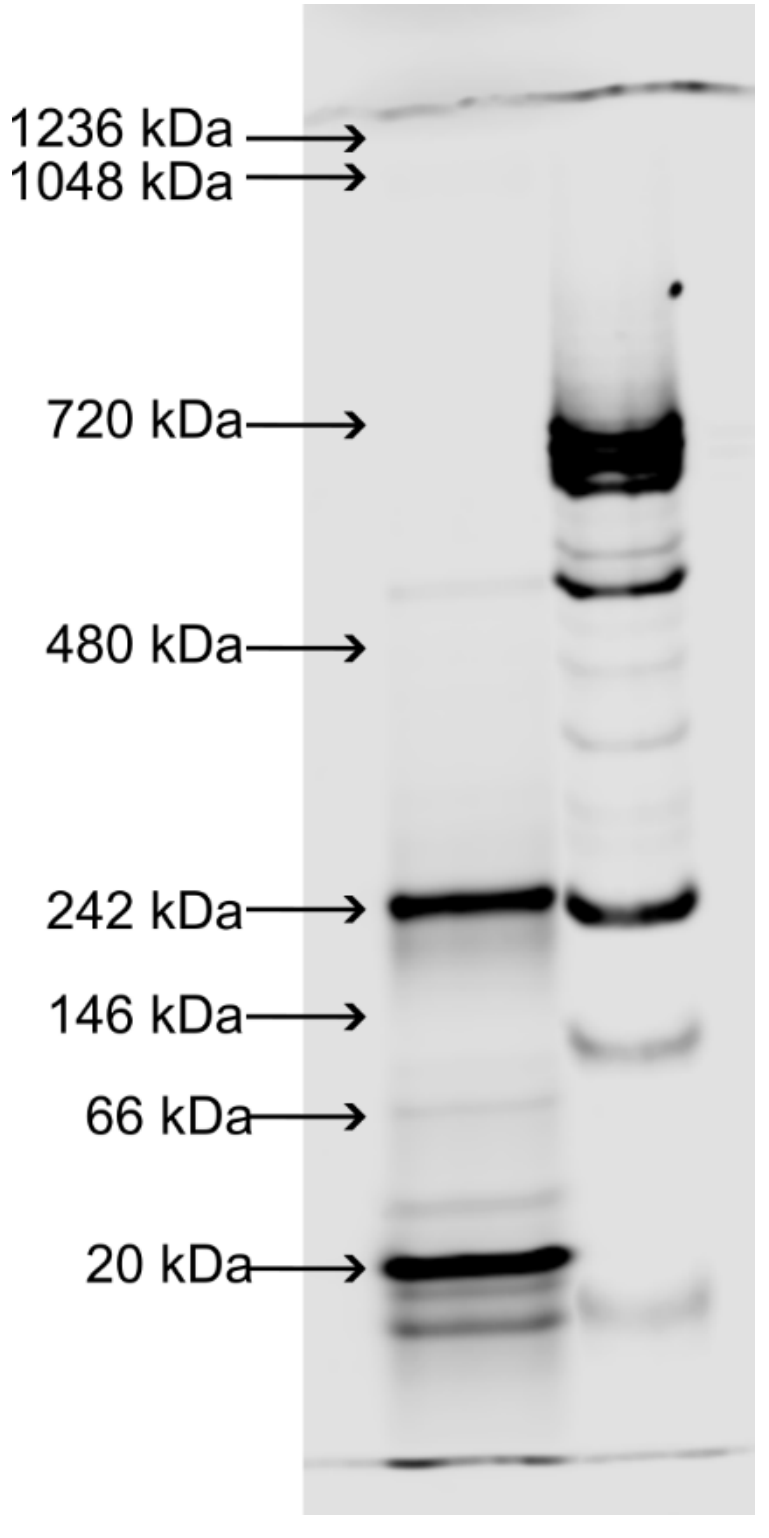

Figure S3 - Sample S1 protein complexes separated using High-Resolution Clear-Native (Hr-CN) PAGE. The gel (4-16%) was cast in-house using the protocol detailed in (57). A total of 540 ng chlorophyll *a* equivalent sample was loaded on the gel. Five µL of the NativeMark™ Unstained protein standard (Invitrogen™) was run alongside the sample. The image shown is the fluorescent image of the unstained gel using the 700 nm channel on the LICOR Odyssey FX imager. The ladder information was gathered by staining the same gel with One-Step Blue® protein gel stain. Gel was run at 200 V for 90 minutes, followed by 350 V for ~4 hours, until the loading Dye of the protein ladder reached the end of the gel. Glycerol was added to the sample to a final concentration of 25%.

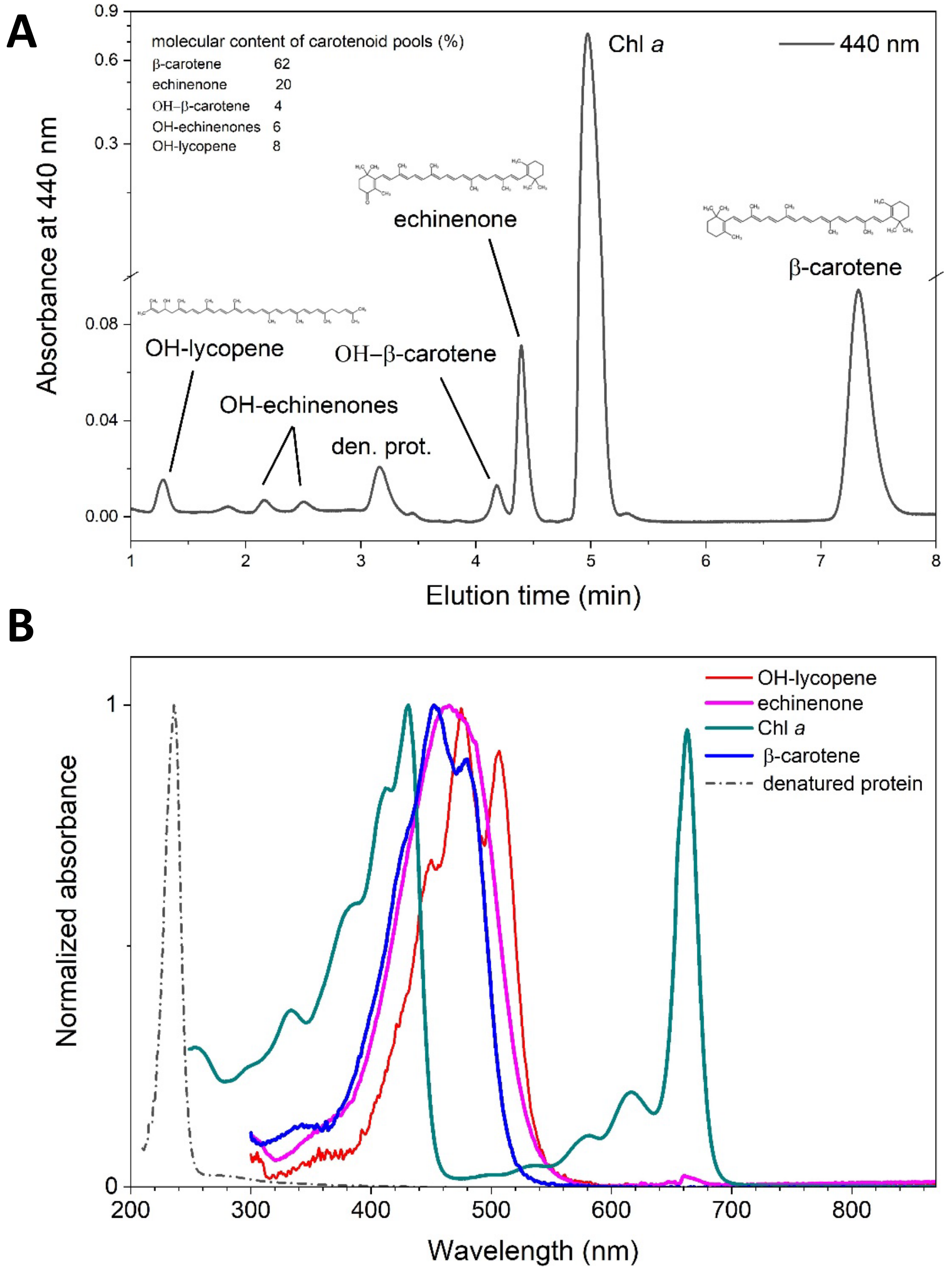

Figure S4 – Pigment content of IsiA-PSI. A. HPLC trace recorded at 440 nm and pigment determination according to spectral appearances and elution times. Note that only oxygenated derivatives or precursors of *β*-carotene are expected. The structures of main carotenoids are provided. The % content of each carotenoid in the entire pool of carotenoids (upper left corner) was calculated according to procedure provided in detail below. Note log scale of absorbance axis above the break. **B**. Absorption spectra of major pigments in IsiA-PSI, normalized at maximum for improved comparability. The minor carotenoids were omitted due to noisy spectral profiles.

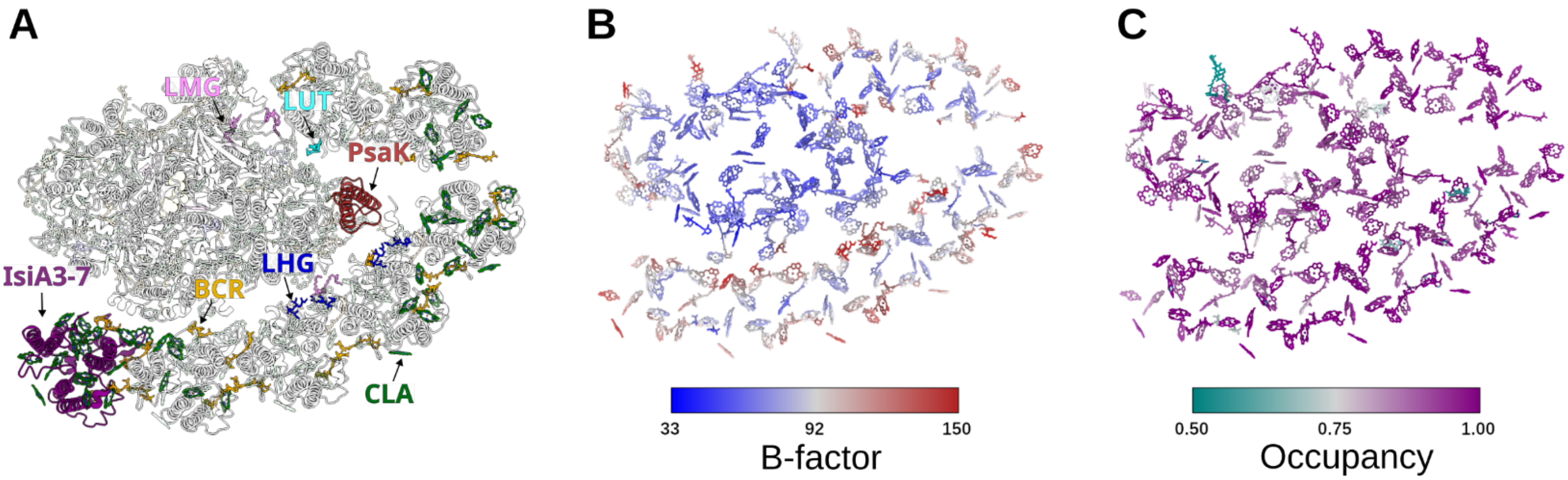

Figure S5 - Highlights of some differences between current and previous model (PDB: 7Y3F). A. In total, 45 Chlorophyll a (CLA, in green) , 19 β-carotene (BCR, in yellow), 3 1,2-distearoyl-monogalactosyl-diglyceride (LMG, in pink) and 4 2-dipalmitoyl-phosphatidyl-glycerol (LHG, in blue) molecules were added in the current model in addition to the modelling of PsaK, the new IsiA3-7 subunits, and the identification of other IsiA gene products. **B & C**. B-factor and Occupancy of chlorophylls, carotene, and lipids in final model of PSI-IsiA from 7120. B-factors were obtained using adp refinement in phenix.refine. Occupancy refinement was likewise performed per-residue with phenix.refine.

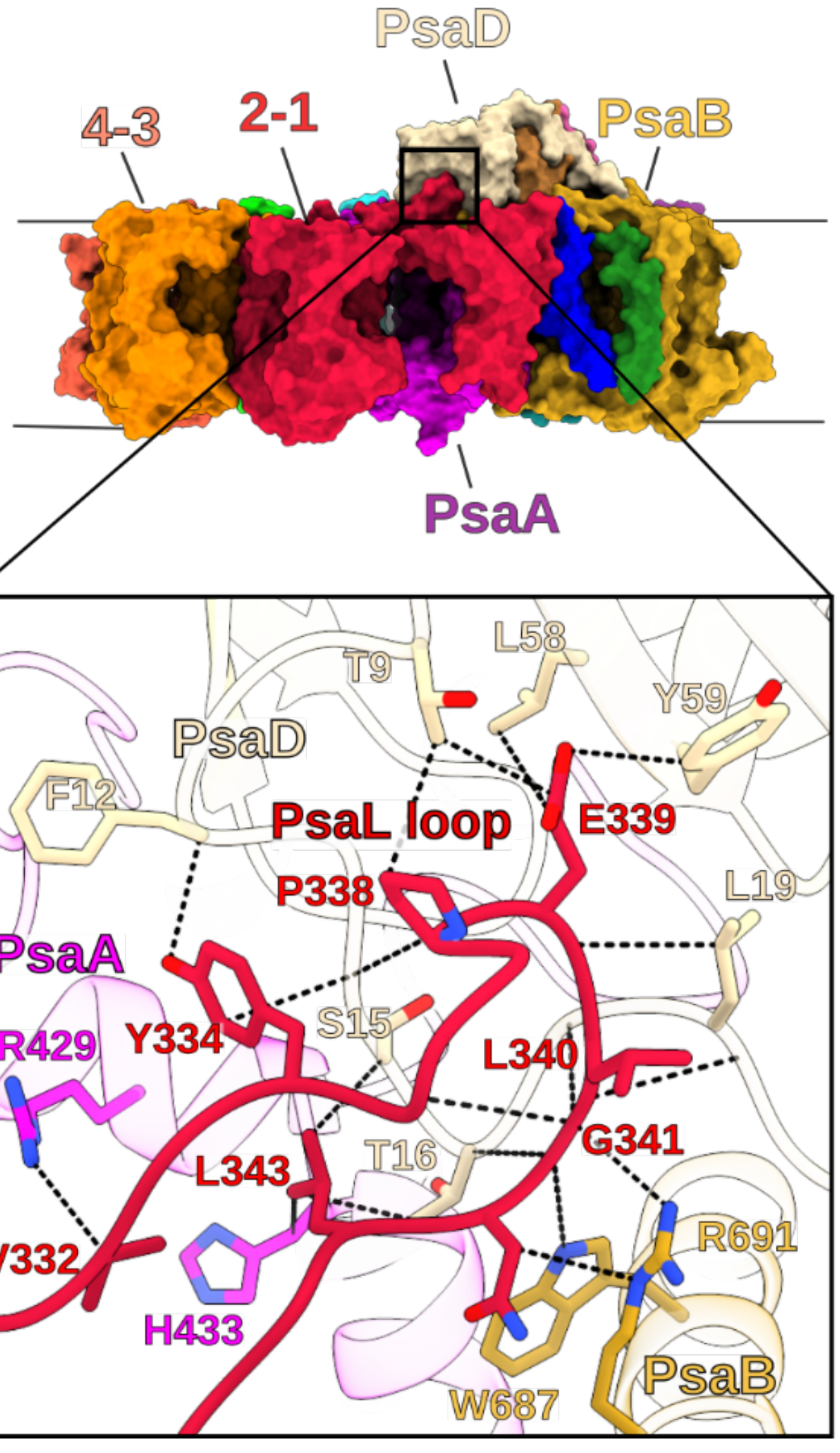

Figure S6 – Interactions between IsiA2-1 PsaL loop and the stromal ridge. Side view of the PSI-IsiA supercomplex honing in on binding cleft of the IsiA/PsaL connecting loop of IsiA2-1 in PSI and the residues that form the interface. This loop interacts with PsaA/D/B of PSI and its structure is conserved from the PsaL subunit.

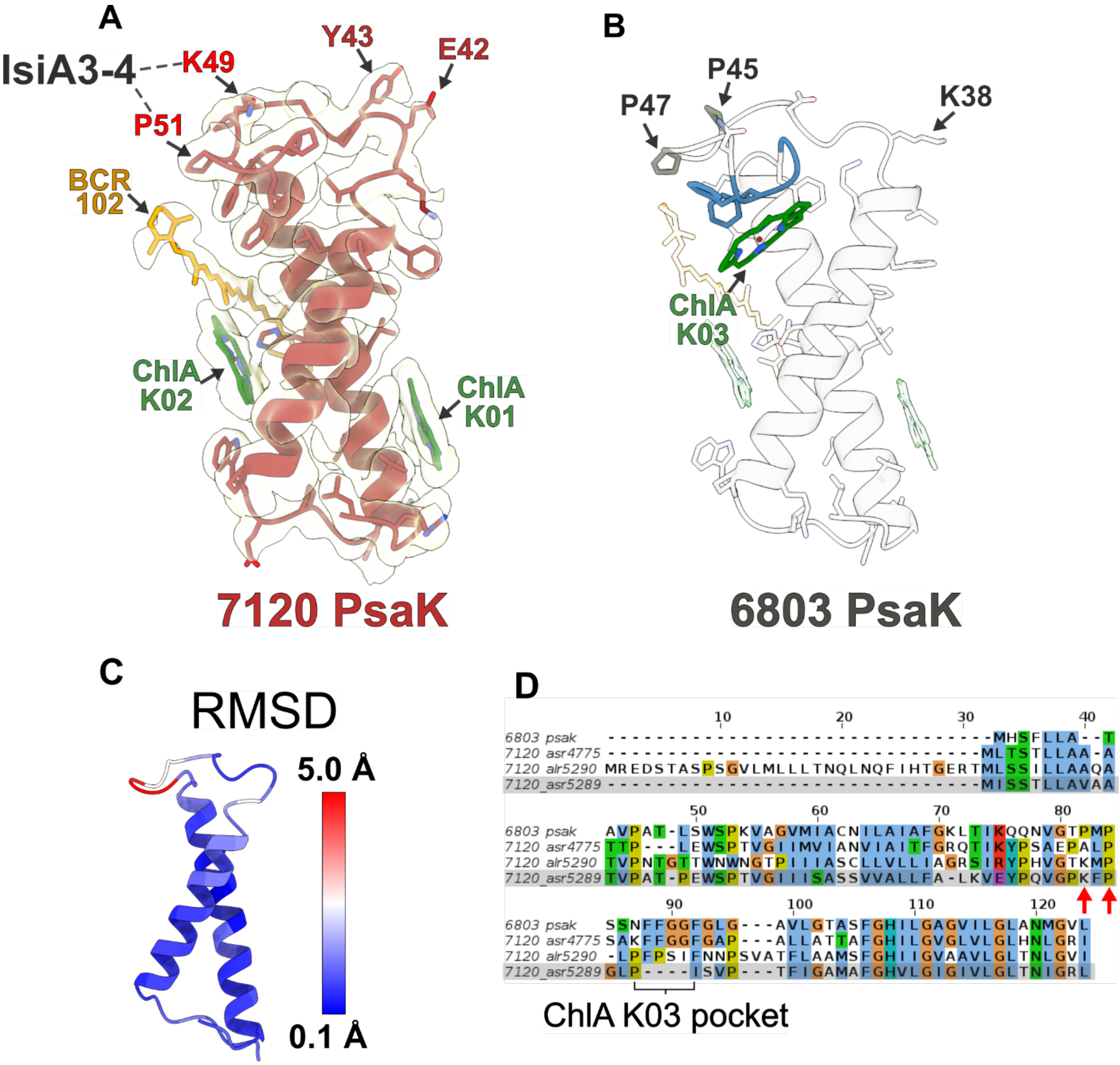

Figure S7 - Side by side comparison of the structure and sequence of PsaK from 7120 and 6803. A. the PsaK gene product (ID: asr5289) and the corresponding density used to model it. This PsaK gene is different from the tetrameric one (asr4775). **B**. the PsaK (PDB: 7UMH) structure from 6803 which possesses ChlA K03 that's missing in the iron complex PsaK. **C**. Per-residue backbone RMSD (red-blue color gradient) of the two structures in 'A' and 'B'. **D**. MSA of the different PsaK genes in 7120 with the one identified in this paper highlighted in gray. The two residues that interact with IsiA3-4 are identified with red arrows. The 6803 sequence is included for reference.

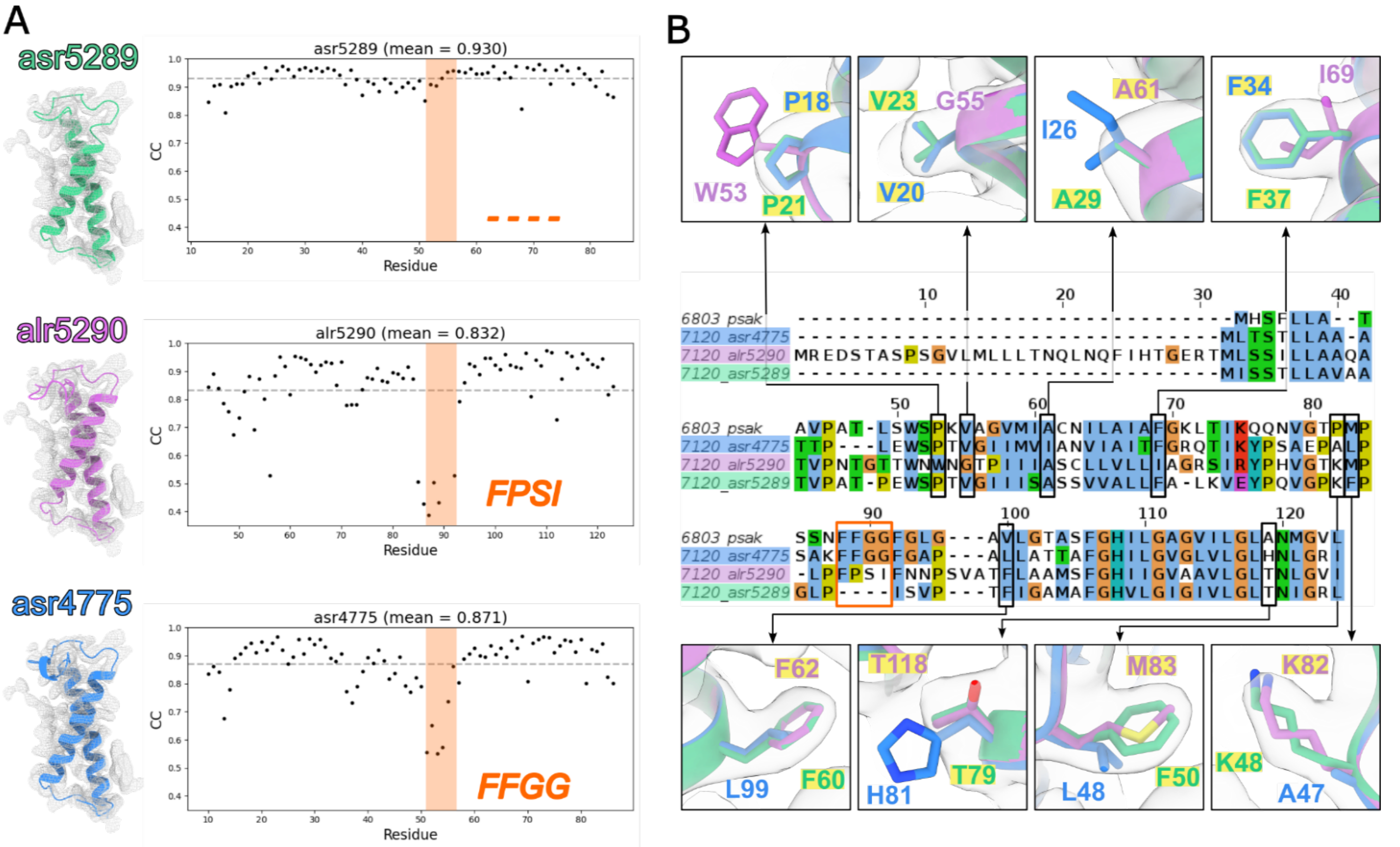

Figure S8 – Verification of PsaK sequence fit in modelled structure. A. Alphafold models of the three PsaK genes in 7120 – asr5289 (green), alr5290 (purple), asr4775 (blue) – and their respective per-residue correlation coefficient (CC) fits after a manual refinement in Coot. The "FFGG" motif is highlighted in orange and is poorly fitted in the experimental density for alr5290 and asr4775. **B**. Diagnostic residues used for sequence discrimination shown in the experimental density. Sequence alignment of the PsaK genes in "A" with the 6803 psak shown as a reference.

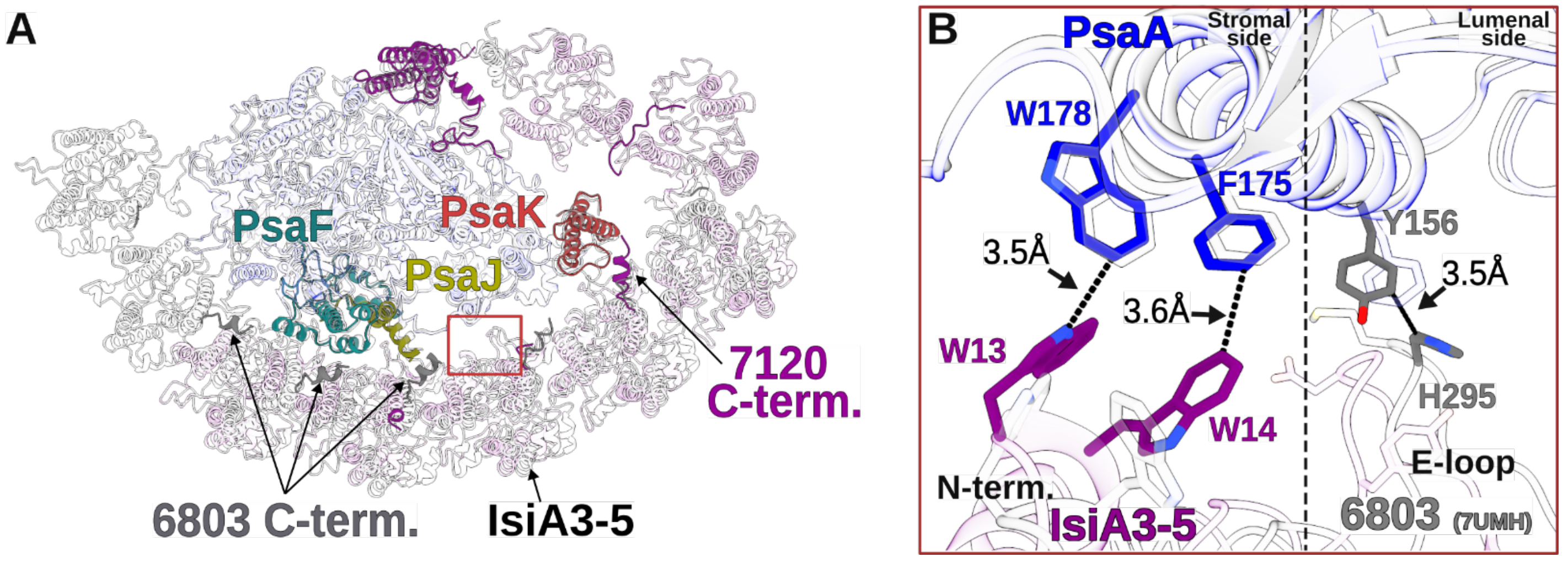

Figure S9 - Differences in IsiA-PSI binding interactions between the 7120 and 6803 complexes. **A**. Superposition of the PSI-IsiA structure from 6803 onto the 7120 model by alignment of the PSI core. PSI proteins that interface IsiA and the IsiA C-termini which are known to anchor the ring onto the PSI core are shown in opaque and labeled. The C-terminus is well folded and interacts specifically at different locations in the two complexes. **B**. Close up view of the interactions at the IsiA3-5 position. At the stromal side of 7120 (left), the aromatic residues W13/W14 of IsiA3-5 are closer to the PsaA W178 and F175 compared to the 6803 structure (PDB: 7UMH, in gray). On the other hand, one significant interaction exists in 6803 at the luminal interface (right) between H295 and Y156 that is absent in 7120.

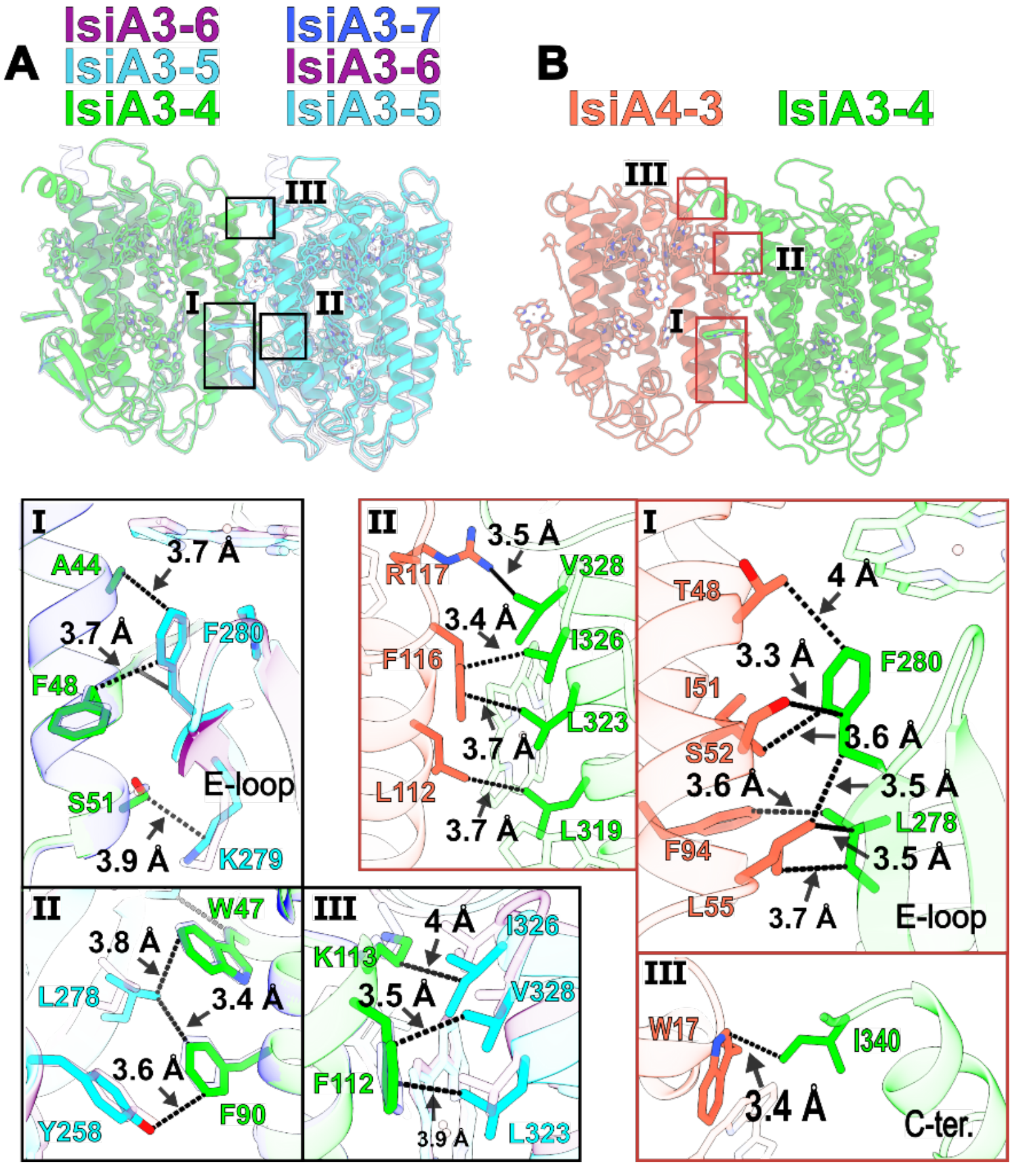

Figure S10 – Additional overview of IsiA-IsiA interactions in the PSI-IsiA supercomplex of 7120. A. Alignment of three different dimeric interfaces composed of IsiA3 with close up views of interaction patches. **B.** The IsiA4-3/3-4 dimer with focused view of interaction patches between the two IsiA subunits.

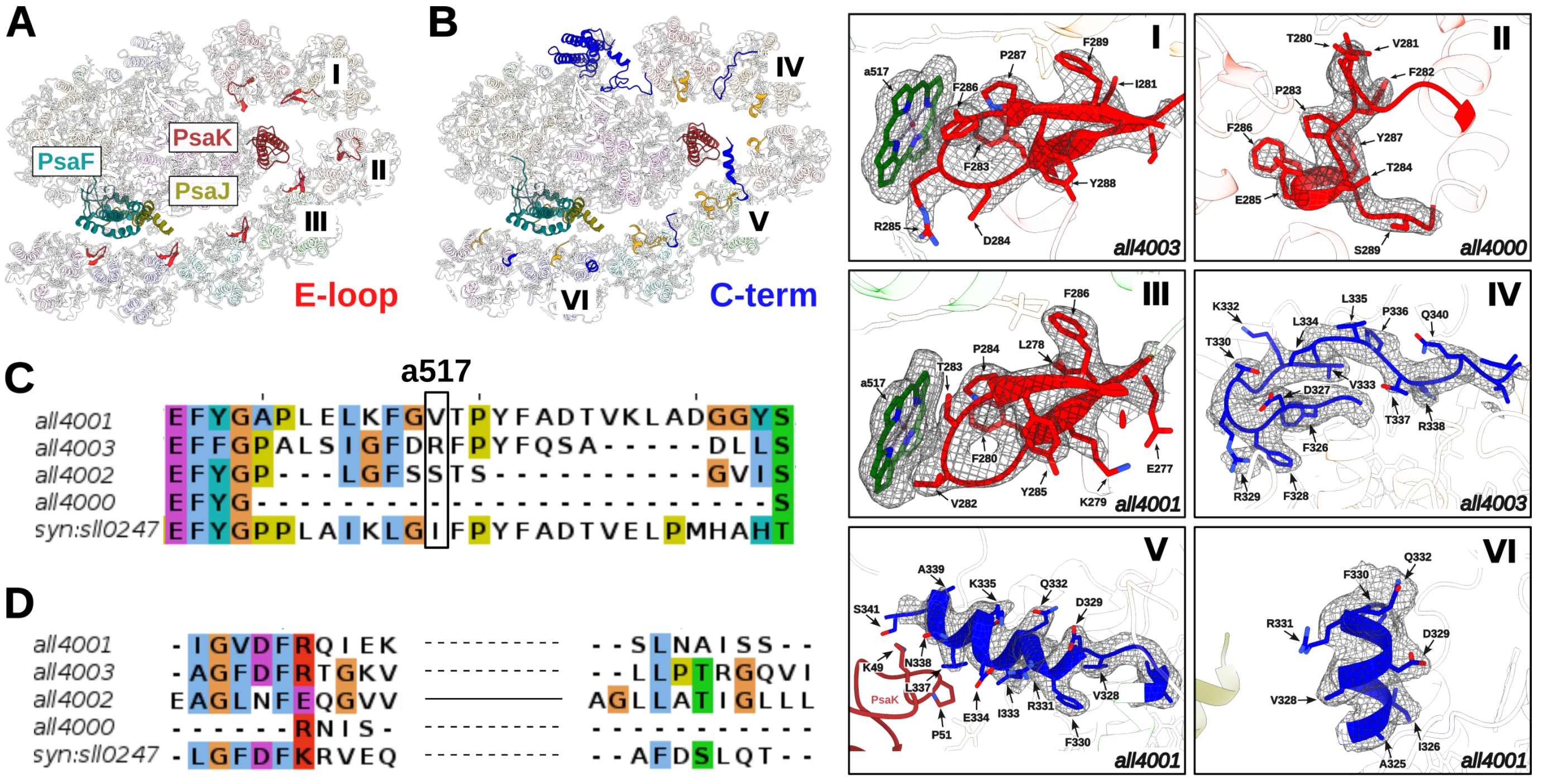

Figure S11 – Structural motifs in the PSI-IsiA supercomplex of *Anabaena sp. PCC 7120*. A. stromal view of the complex with the IsiA E-loop amino acid sequences colored in red . PSI subunits PsaF/J/K of the core are highlighted. **B**. lumenal view of the overall complex with C and N termini of each IsiA colored in blue and gold respectively. **C-D.** Multiple Sequence Alignment of different IsiA E-loop and C terminus sequences in *Anabaena sp. PCC 7120.* The *Synechocystis sp. PCC 6803* sequence (syn:sll0247) is shown as reference. Subpanels I-III show close up views of the chlorophyll 517 coordinating E-loop regions (sequence box in "C".) of the atomic model for select IsiA subunits. Amino acid side chains are shown and labeled in each view and the corresponding density data is shown in mesh. Subpanels IV-VI show the C-terminus model and map for subunits denoted in "B" revealing different sequence and conformations of the IsiA C-terminus in the super complex.

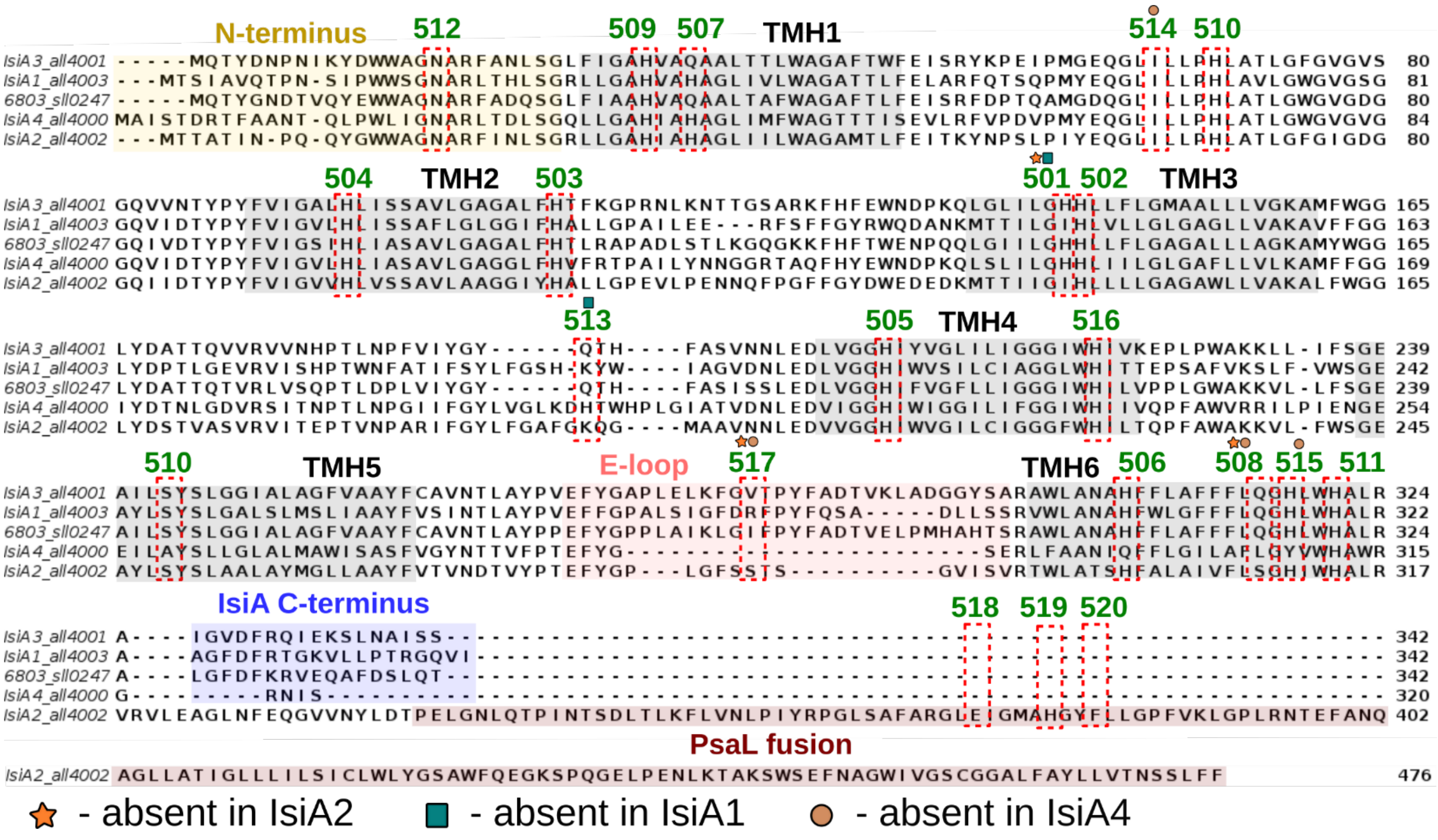

Supplementary Figure 12 - Multiple sequence alignment and annotation of IsiA genes. The alignment was performed using genes from *Anabaena sp. PCC 7120* (all4000-all4003) and the IsiA gene from *Synechocystis sp. PCC 6803* (sll0247) with MUSCLE and visualized with Jalview. Sequences were annotated to highlight N-termini, transmembrane domains (TMH), C termini, the PsaL fusion part of IsiA2-1, and chlorophyll coordinating residues (in green). Certain chlorophyll that are absent in different IsiA structures are also denoted with symbols.

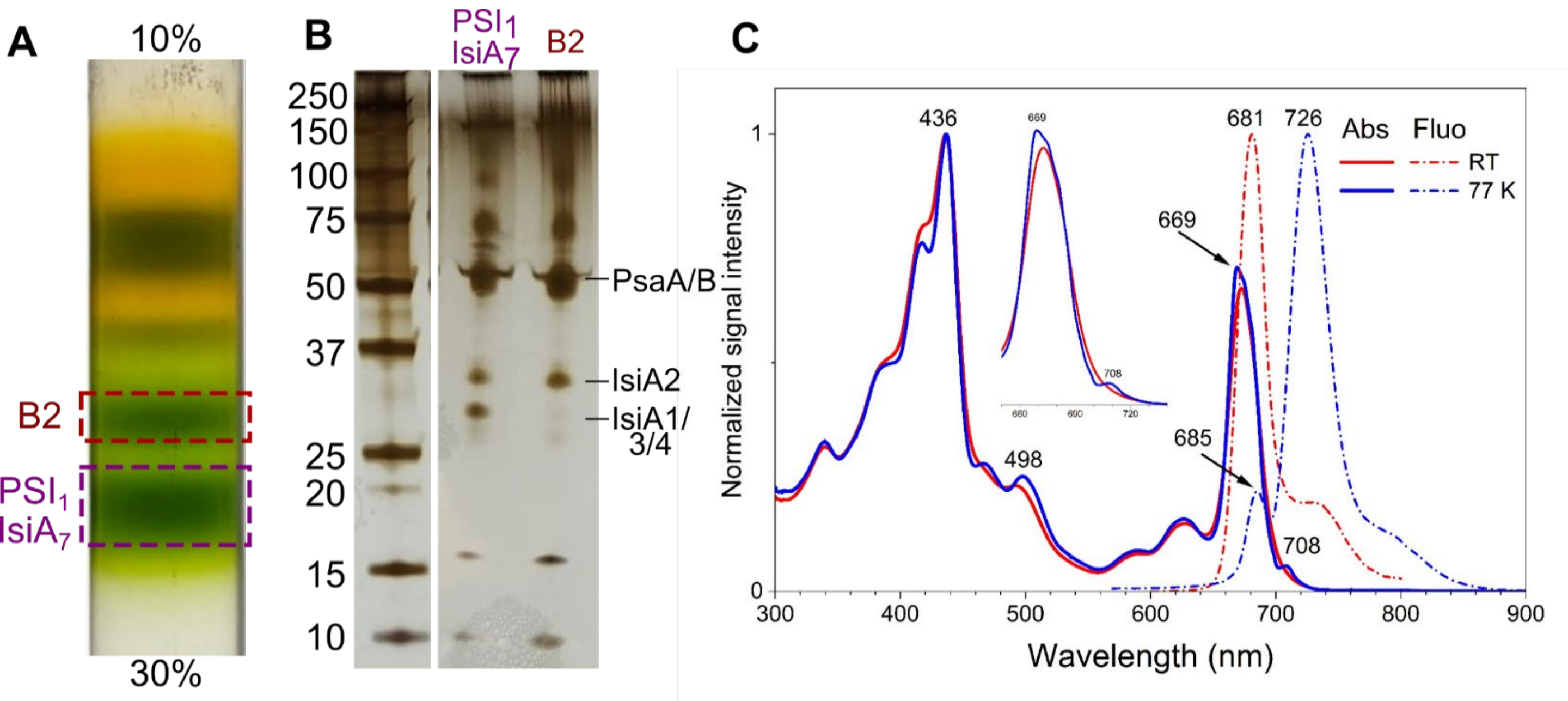

Figure S13 – Separation, content and basic spectroscopic properties of IsiA-PSI. A. 10-30% Sucrose gradient of solubilized thylakoid membranes with the high molecular weight green band used for imaging and spectroscopic characterization highlighted in purple box. Another green band B2 (in maroon) was also observed. **B**. sodium dodecyl sulfate–polyacrylamide gel electrophoresis of the bands in "A" was performed to separate proteins on a 4–20 % bis-tris precast gel (SurePAGE™, Bis-Tris, 10×8, 4-20%, 15 wells; GenScript) according to the manufacturer's instructions. Bands corresponding to the core PSI subunits (PsaA/B) and IsiAs are highlighted. **C**. Absorption and fluorescence emission spectra of IsiA-PSI (PSI_1-$IsiA_7$ band) at room temperature and at 77 K. For better comparability all spectra were normalized at their maxima and all wavelengths of all major bands are indicated.

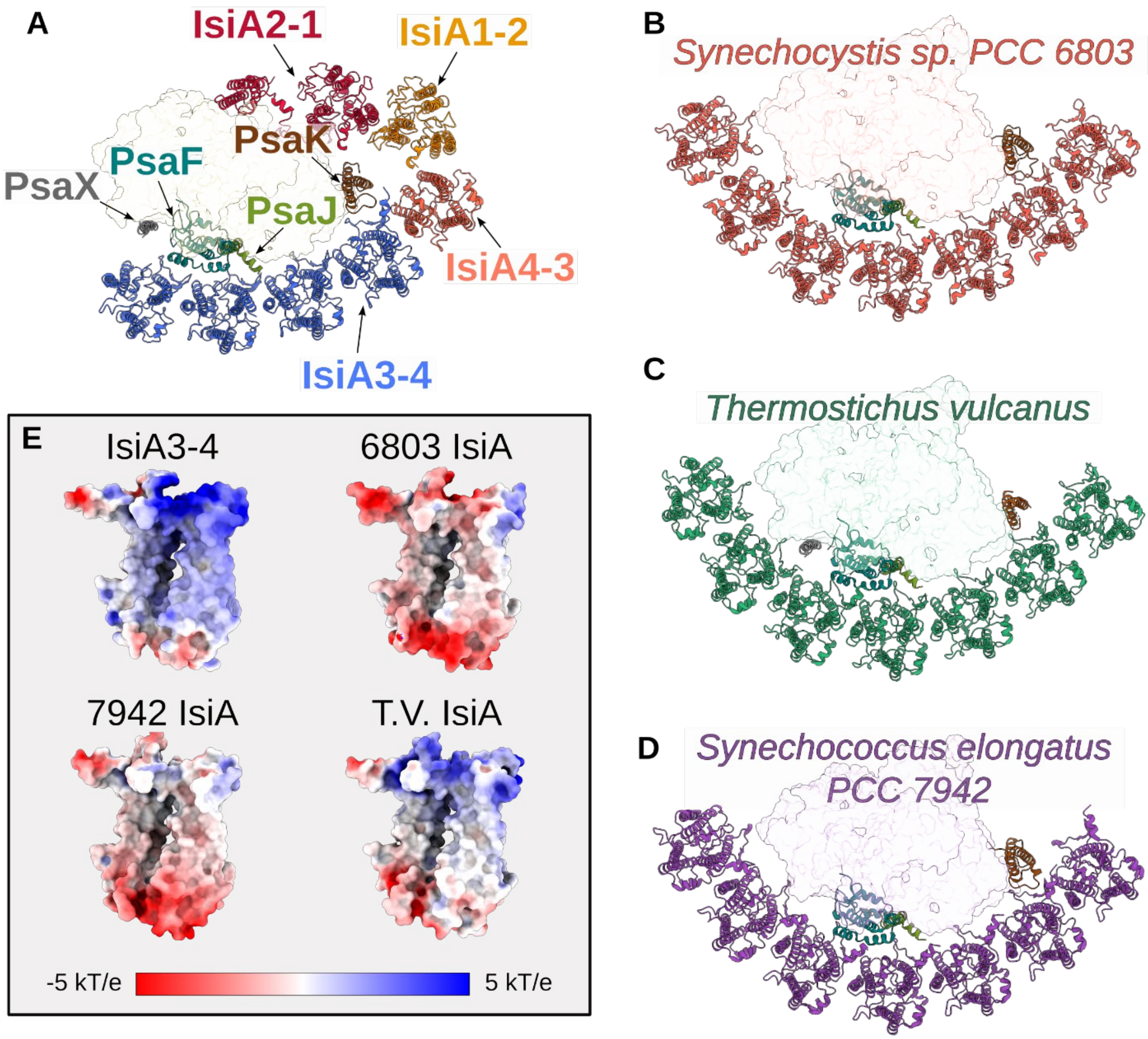

Figure S14 - PSI-IsiA complexes in different cyanobacterial species. A. the 7120 complex with 4 different IsiA genes (IsiA3 in blue, IsiA4 in salmon, IsiA1 in orange, IsiA2 in red) and the interfacing PsaF/J/K in opaque. **B-D.** Approximately one third of PSI_3-$IsiA_{18}$ complex from 6803, T.V., and 7942 are shown. **E.** Electrostatic potential surfaces of the IsiA subunits from different structures in "A-D" calculated using Atomic Poisson Boltzman solver webtool showing differences that may explain variation PSI binding in the different structures *(55).*

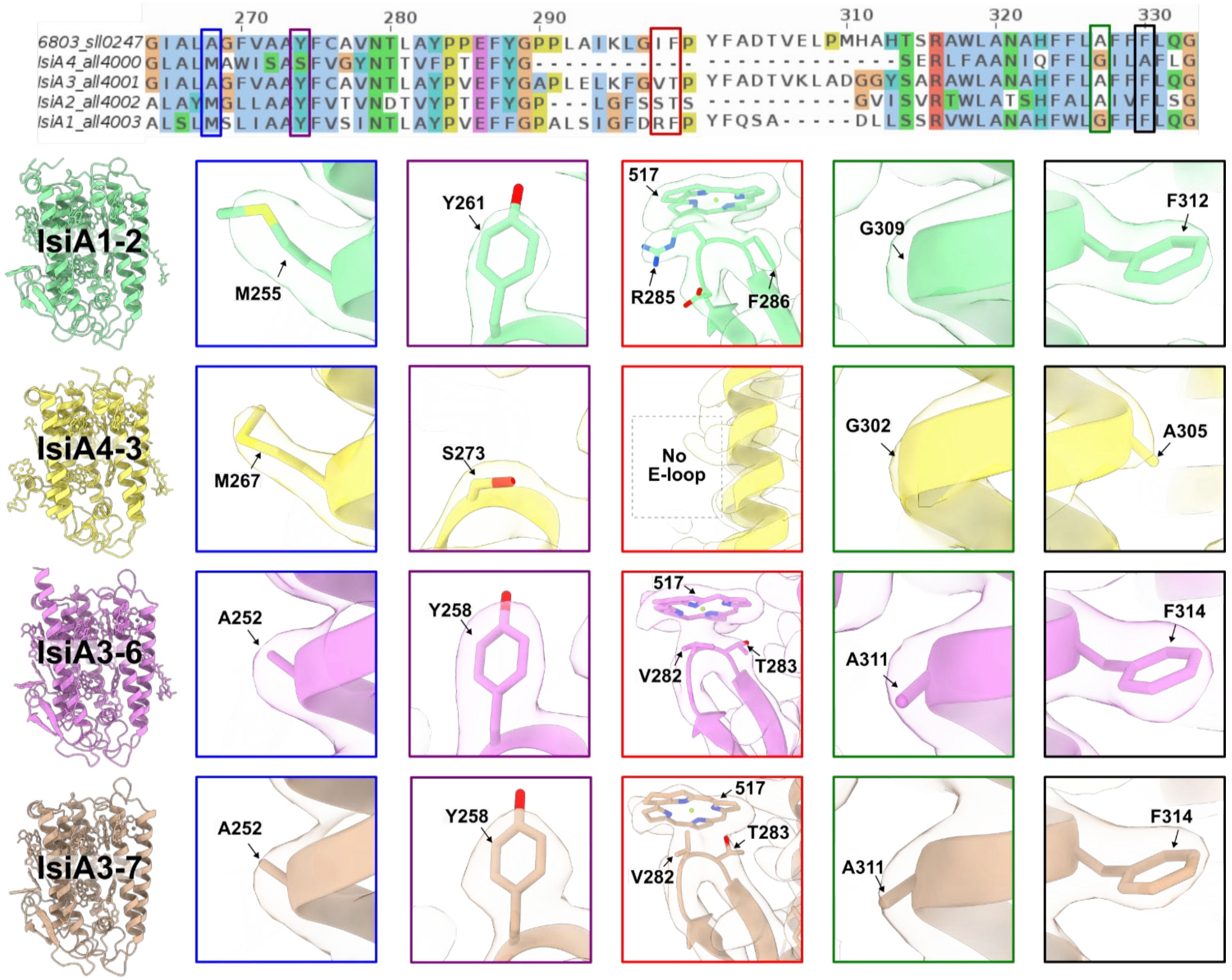

Figure S15 – Diagnostic residues used for assignment of IsiA sequences to previously un-modeled subunits. Panels of side chain residues shown fitted into the experimental density for the 4 newly modeled IsiA subunits in this study: IsiA1-2 (green), IsiA4-3 (yellow), IsiA3-6 (pink), and IsiA3-7 (brown).

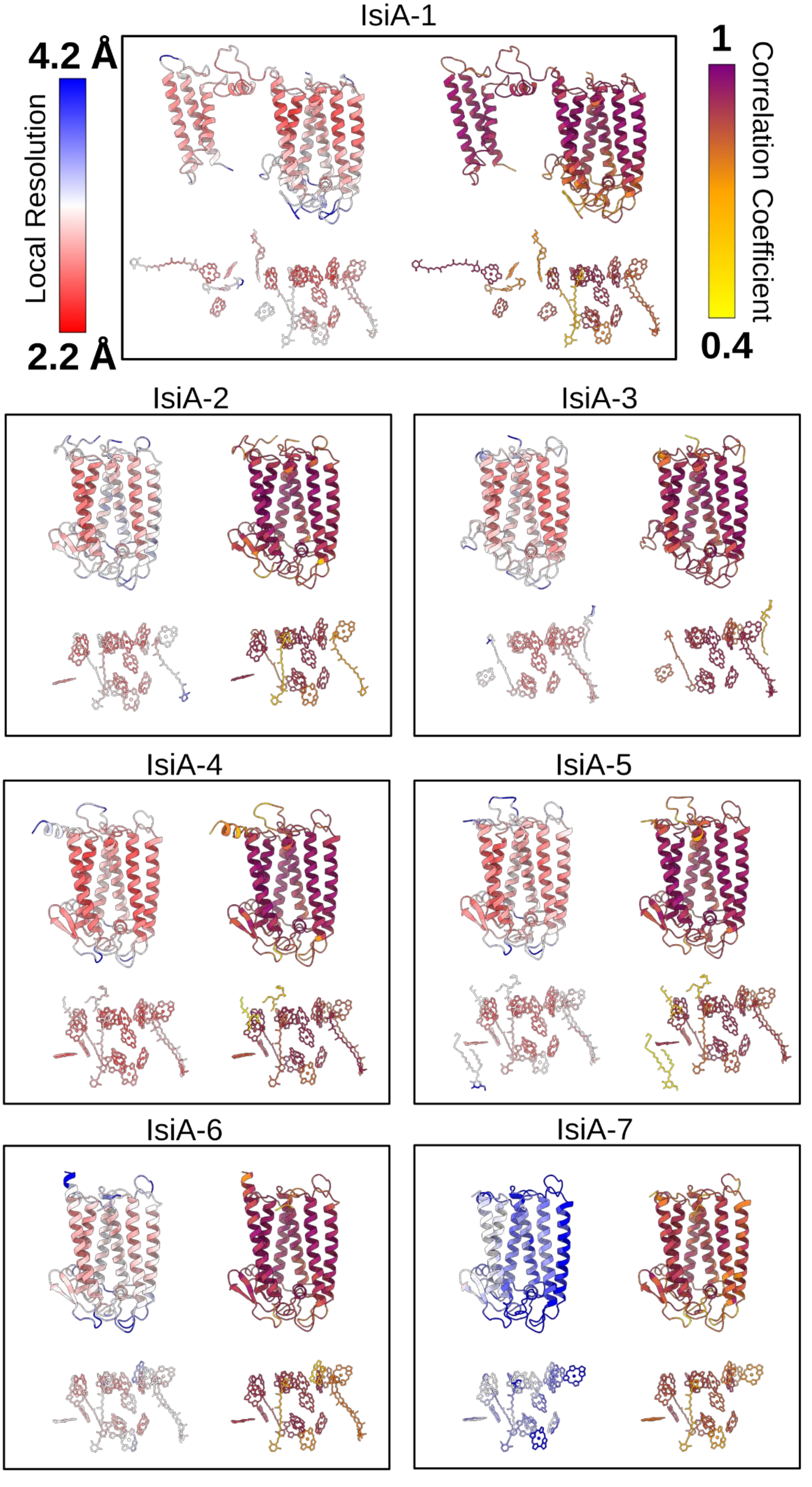

Figure S16 – Local resolution and correlation coefficient panels for each IsiA subunit. Local resolution maps obtained from cryoSPARC “local resolution” jobs were used in Chimerax to interpolate values onto each atom via the “measure mapvalues” function. The correlation coeficients are direct outputs from phenix.refine.

	PSI-IsiA7 (EMD-74341, PDB 9ZJT)
**Data collection and processing**	
Magnification	165,000
Voltage (kV)	300
Electron exposure ($e^{-}/Å^{2}$)	35
Defocus range (μm)	0.8 – 2.5
Pixel size (Å)	0.731
Symmetry imposed	C1
Initial particle images (no.)	975,718
Final particle images* (no.)	96,842
Map resolution (Å)	2.22
FSC threshold	0.143
**Refinement**	
Initial model used (PDB code)	7Y3F
Model resolution (Å)	2.13/2.48
FSC threshold	0.143/0.5
Map sharpening *B* factor ($Å^{2}$)	See methods
Model composition	
Nonhydrogen atoms	49,418
Protein residues	4513
Ligands	274
B factors ($Å^{2}$)	
Protein	93.30
Ligand	88.90
R.m.s. deviations	
Bond lengths (Å)	0.003
Bond angles (°)	0.849
**Validation**	
MolProbity score	0.87
Clashscore	1.10
Poor rotamers (%)	0.31
Ramachandran plot	
Favored (%)	97.74
Allowed (%)	2.26
Disallowed (%)	0

Table S1 - Data collection and refinement statistics.

PSI$_1$-IsiA$_7$ from 7120 (PDB: 9ZJT)					
PSI chain	Res ID	IsiA chain	Res ID	Rate (ps^{-1})	κ^2
J	1303	6	514	1.32	2.69
A	1801	1	511	0.65	0.99
J	1303	7	517	0.59	0.19
A	1121	1	511	0.36	2.64
J	1302	6	517	0.26	0.55
K	1401	2	517	0.17	3.89
J	1303	7	508	0.16	1.12
K	1401	1	514	0.12	0.91
A	1105	6	517	0.12	2.17
A	1108	5	511	0.1	3.09
A	1114	5	517	0.09	0.18
A	1105	5	514	0.09	2.47

One third PSI$_3$-IsiA$_{18}$ from 6803 (PDB: 7UMH)					
PSI chain	Res ID	IsiA chain	Res ID	Rate (ps^{-1})	κ^2
K	4003	g	517	0.23	1.84
J	1303	Y	514	0.18	2.21
K	4003	g	508	0.15	0.38
F	1302	X	514	0.13	1.83
B	1232	W	514	0.1	1.4
A	1114	Z	517	0.08	0.33
A	1108	Z	511	0.08	3.34

Table S2 - FRET rates and orientation factors (κ^2) between PSI and IsiA chlorophyll a pigments. Rates for only one third of the PSI$_3$-IsiA$_{18}$ complex from 6803 are shown which involved an IsiA hexamer and a PSI monomer. Only rates that are below a lifetime threshold of 12.5 ps are listed. κ^2 refers to the Forster orientation factor which was calculated as expressed in the methods.

Chain	Atom inclusion	Q-score
All	0.9040	0.5950
1	0.8670	0.5670
2	0.8270	0.5480
3	0.8560	0.5600
4	0.8830	0.5690
5	0.8750	0.5580
6	0.9130	0.5280
7	0.7690	0.4610
A	0.9670	0.6610
B	0.9560	0.6520
C	0.9850	0.6620
D	0.9530	0.6440
E	0.9350	0.6350
F	0.9390	0.6410
I	0.9370	0.6230
J	0.9600	0.6390
K	0.9110	0.6230
M	0.9520	0.6390
X	0.9280	0.6300

Table S3 – Map to model fit summary. Average atom inclusion at the contour level (σ=4.34) and Q-score for the entire model and for each chain.

Data S1: Ligands B-factor and occupancy values for PSI-IsiA.

ligand_Bfactor_occupancy_cc_summary.xlsx
